# G-quadruplex induced chirality of methylazacalix[6]pyridine via unprecedented binding stoichiometry: en route to multiplex controlled molecular switch

**DOI:** 10.1038/srep10479

**Published:** 2015-05-20

**Authors:** Ai-Jiao Guan, Meng-Jie Shen, Jun-Feng Xiang, En-Xuan Zhang, Qian Li, Hong-Xia Sun, Li-Xia Wang, Guang-Zhi Xu, Ya-Lin Tang, Li-Jin Xu, Han-Yuan Gong

**Affiliations:** 1 Beijing National Laboratory for Molecular Sciences, Center for Molecular Sciences, State Key Laboratory for Structural Chemistry of Unstable and Stable Species, Institute of Chemistry, Chinese Academy of Sciences, Beijing, 100190 (P. R. China); 2 Department of Chemistry, Renmin University of China, Beijing, 100872 (P. R. China); 3 College of Chemistry, Beijing Normal University, Beijing, 100875 (P. R. China)

## Abstract

Nucleic acid based molecular device is a developing research field which attracts great interests in material for building machinelike nanodevices. G-quadruplex, as a new type of DNA secondary structures, can be harnessed to construct molecular device owing to its rich structural polymorphism. Herein, we developed a switching system based on G-quadruplexes and methylazacalix[6]pyridine (MACP6). The induced circular dichroism (CD) signal of MACP6 was used to monitor the switch controlled by temperature or pH value. Furthermore, the CD titration, Job-plot, variable temperature CD and ^1^H-NMR experiments not only confirmed the binding mode between MACP6 and G-quadruplex, but also explained the difference switching effect of MACP6 and various G-quadruplexes. The established strategy has the potential to be used as the chiral probe for specific G-quadruplex recognition.

Molecular device design and construction have seen great achievement in the past three decades^1^,^2^,^3^,^4^,^5^,^6^,^7^,^8^,^9^,^10^,^11^,^12^. Introduction of DNA to form molecular device (e.g., molecular switch) has been approved as an effective approach owing to its structural polymorphism and molecular recognition ability^13^,^14^,^15^,^16^,^17^. Since the secondary and tertiary structure of DNA can be manipulated via sequence design, it provide a powerful approach for the construction of molecular device^18^,^19^,^20^,^21^. Compared to the traditional nucleotides, G-quadruplex, which formed from guanine-rich oligonucleotide sequences, has attracted a growing interest in application of nanodevice and molecular switching^22^,^23^,^24^,^25^,^26^. To date, G-quadruplex involved switching systems based on the characterized signal of G-quadruplex^27^,^28^,^29^, or mediated by iron^30^,^31^,^32^,^33^,^34^,^35^ or fluorescent probe^36^,^37^,^38^,^39^,^40^ have been reported. These works showed the G-quadruplex had great potential in construction of molecular device. However, there are very few reports, if any, of which we know that the stimuli responsive chiral switching system of small molecule mediated by G-quadruplex.

We show herein the construction of a stimuli responsive molecular switch controlled via temperature as well as pH characterized by the induced CD signal of MACP6 (Fig. 1) and G-quadruplex. The detailed investigation of the binding mechanism between MACP6 and a series of G-quadruplexes (i.e. c-kitG20T, c-mycTGA-de or c-myc1245) (see Table S1) via circular CD spectroscopy, ^1^H-NMR spectra and molecular modeling was carried out.

In our previous studies, the chirality of MACP6 could be induced by a series of G-quadruplexes^41^, and showed cotton effect (CE) dependence on the configuration of G-quadruplexes owing to the conformation-specific recognition property^42^. These findings suggested that the CD signal of MACP6 could characterize the interaction between G-quadruplex and MACP6, as well as the response of the complex to the environmental stimuli shown below.

## Results and Discussion

### Switching controlled by temperature

**Figure 2:**
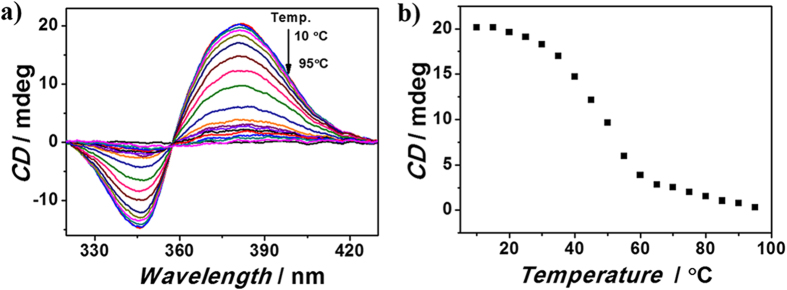
**a**) The CD spectra, and **b**) the CD intensity (380 nm)/temperature profile of the mixture containing MACP6 (10 μM) and c-kitG20T (20 μM) corresponding to different temperatures from 10 °C to 95 °C in 17 mM phosphate buffer solution (5% DMSO) at pH 7.40.

Oligonucleotide c-kitG20T^41^,^43^, c-mycTGA-de^41^,^44^ and c-myc1245^41^,^45^ can form intramolecular parallel G-quadrupelx with difference in the loops in phosphate buffer solution. As expected, given their binding nature via non-covalent weak interaction, the complexes between MACP6 and G-quadruplex (c-kitG20T, c-mycTGA-de or c-myc1245) were found to be sensitive to environmental conditions. Herein, we demonstrate the stimuli effect of the complexes formed between MACP6 and G-quadruplex of c-kitG20T, c-mycTGA-de or c-myc1245, respectively. A positive CE of MACP6 was observed when complexed with G-quadruplex in the wavelength scale from 320 nm to 430 nm at room temperature (20 °C) (see Figure S1-S3). The positive peak value at 380 nm of MACP6 was adopted here to evaluate the binding property between MACP6 and G-quadruplex at various temperatures. Equilibrium state of MACP6/G4 was determined from 10 to 95 °C by every five degree via recording the CD spectra of the complex at its steady state (Fig. 2a and Figure S4-S5). The results were rearranged to a CD intensity (380 nm)/temperature profile (Fig. 2b). In the case of MACP6/c-kit G20T, CD signal at 380 nm maintained similar positive values below 25 °C (from 20.1 mdegree at 10 °C to 19.1 mdegree at 25 °C). With the temperature increased, the CD signal decreased sharply to 2.8 mdegree at 60 °C, and remained unchanged with further heating until 95 °C. The downward curve from 25 °C to 60 °C indicated nonequilibrium process of the complexing and decomposing state between MACP6 and c-kitG20T (Fig. 2b). Following the above mentioned experimental procedure, the complexation or decomposed states of mycTGA-de/MACP6 has been determined to be below 45 °C or above 65 °C (see Figure S6). Meanwhile, it was found that the temperature lower than 40 °C or higher than 65 °C corresponded to complexation or dissociation state of c-myc1245 and MACP6 (see Figure S7).

**Figure 3:**
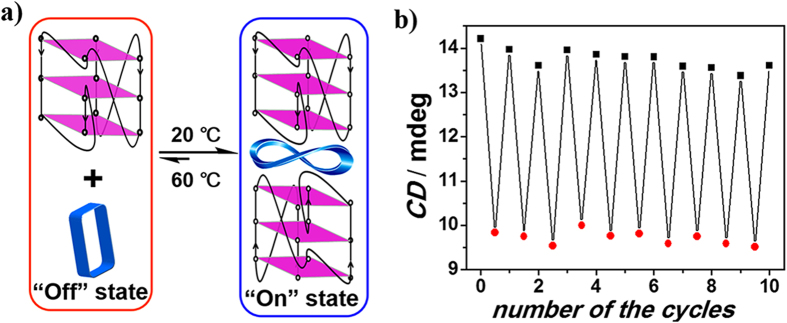
Schematic (**a**) and graphical representation (**b**) of the complexation and dissociation of MACP6/c-kitG20T as inferred from temperature dependent CD spectroscopic studies. Here, CD signal changes at 380 nm of MACP6 (4 μM) and c-kitG20T (8 μM) in 17 mM phosphate buffer solution (5% DMSO) at 20 °C (represented as“⚬”) and 60 °C (represented as“•”) were used to monitor the switching “on” and “off” of the molecular switch.

Accordingly, the initial temperature-response of the complexation between MACP6 and c-kitG20T G-quadruplex was carried out. Temperature increment led to reduced CE of MACP6, characterized with the CD value at 380 nm as 14.2 and 9.2 mdegree at 20 °C and 60 °C, respectively (Fig. 3b). When the temperature dropped to 20 °C from 60 °C and then stayed for 3 min, the CD spectra backed to the original state before heating, namely strong CE effect from 320 nm to 430 nm, with peak value as 13.6 mdegree at 380 nm. It is noted that even after ten cycles, the switch still works well as the first one (Fig. 3). Furthermore, variable temperature CD (Figure S8) and ^1^H-NMR (Figure S9-S10) results confirmed that formation and denaturation of the c-kitG20T G-quadruplex occurred with the temperature cycling, thus resulting in the switchable signal of G4/MACP6 complex.

As c-kitG20T, c-mycTGA-de or c-myc1245 induced analogous positive CE of MACP6, with peak value at 380 nm. The heating-cooling switchable response of both complexes has also been observed. Different with the good stability of temperature-controlled switch formed between MACP6 and c-kitG20T (Fig. 3), the “dampening” of the effect with each cycle has been observed. Specially, the positive peak value at 380 nm of the MACP6/c-mycTGA-de complex reduced 41% (from 17.9 to 10.6 mdegree) after 10 cycles (see Figure S11). It is noted that the binding of MACP6 and c-myc1245 easiest decomposed via heating/cooling cycles, with 81% (from 12.0 to 2.26 mdegree) signal value reduction after 10 cycles (see Figure S12). Although the variable temperature CD and ^1^H-NMR cycling results of mycTGA-de (Figure S13-S15) or c-myc1245 (Figure S16-S18) G-quadruplex showed that the signal of G-quadruplex can be recovered, a high melting point of G-quadrulex of mycTGA-de or c-myc1245 compared to c-kitG20T (Figure S19-S21 and Table S2) decreased the activity of the binding MACP6, thus resulted to a decreased G4 (mycTGA-de or c-myc1245)/MACP6 complex.

### Switching controlled by pH

**Figure 4:**
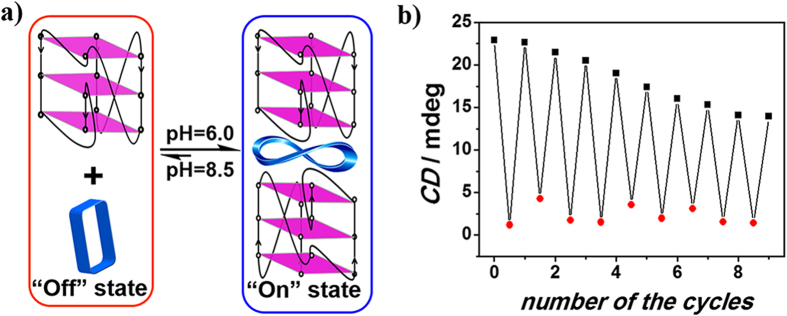
Schematic (**a**) and graphical representation (**b**) of complexation and decomposition of MACP6/c-kitG20T as inferred from pH dependant CD spectroscopic studies. Here, CD signal changes at 380 nm with pH 6.0 (represented as“⚬”) or pH 8.5 (represented as“•”) of MACP6 (10 μM) and c-kitG20T (20 μM) in phosphate buffer solution (5% DMSO) were used to monitor the switching “on” and “off” of the molecular switch. The first step in each cycle is the addition of 0.4 M NaOH solution and the second was the addition of 0.4 M HCl solution, respectively.

Further support for the reversible nature of the switching system formed between MACP6 and G-quadruplex came from pH dependant CD spectroscopic studies. The binding or decomposing state of MACP6 and G-quadruplex (c-kitG20T, c-mycTGA-de or c-myc1245) has been observed at the pH value lower than 7.25 or higher than 8.5 via stepwise CD detection (see Figure S22-S27). MACP6 and c-kitG20T G-quadruplex bonded each other effectively at pH as 6.0 and a positive CE was observed with maximum value as 22.9 mdegree at 380 nm (Fig. 4b). Decreasing the acidity of solution to pH 8.5 via adding 0.4 M NaOH aqueous solution, the CD signal reduced to 1.2 mdegree at 380 nm. Following with adding 0.4 M HCl aqueous solution into the basic system to back pH value as 6, the induced positive CE of MACP6 returned to its original value (Fig. 4b). This process may be repeated with subsequent additions of acid and base serving to “switch on” and “switch off” G4/MACP6 complex. As expected, the increased salt in solution serves to buffer the effect of each new addition of acid or base. Meanwhile, the additional volume of the whole system, even it is small, also contribute to the “dampening” of the effect with each cycle. Nevertheless, it is possible to repeat the complexation and decomposition process several cycles, as can be observed in Fig. 4. The similar pH “switch” was observed in the case of c-mycTGA-de (see Figure S28) or c-myc1245 (see Figure S29). Since the ^1^H-NMR spectra (see Figure S30-S32) showed that the configuration of G-quadrulex kept stable from pH 6.0 to 8.5, it is supposed that MACP6 is more protonated at pH 6.0 than 8.5, and its protonation enhanced the binding with the negatively charged G-quadruplex^46^.

### Circular dichroism spectra and Job-plot for binding stoichiometry

**Figure 5:**
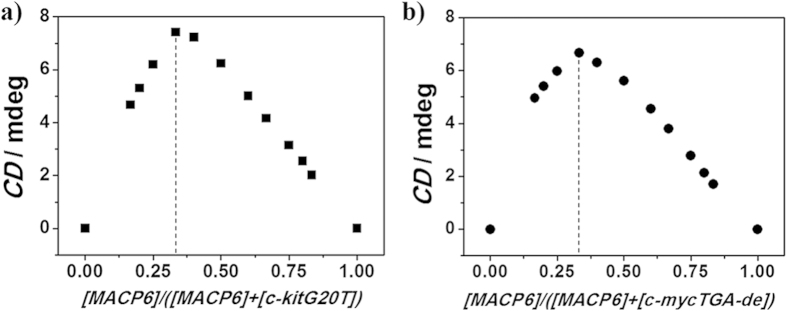
The CD Job-plot corresponding to the binding between MACP6 and G-quadruplex of **a**) c-kitG20T, b) c-mycTGA-de in 17 mM phosphate buffer solution (5% DMSO) at 20 °C. [MACP6] + [G-quadruplex] = 12 μM. The similar maximum value of y (defined as the CD intensity value of the MACP6/G4 complex) at 380 nm was found at 0.33 for c-kitG20T or c-mycTGA-de, a finding consistent with a 1:2 (MACP6: G4) binding stoichiometry.

To understand the switching mechanism, interactions between MACP6 and three G-quadruplexes (c-kitG20T^41^,^43^, c-mycTGA-de^41^,^44^ or c-myc1245^41^,^45^) have been detailed evaluated in 17 mM phosphate buffer solution (K_2_HPO_4_/KH_2_PO_4_ and 5% DMSO, pH 7.40) via CD spectroscopy. The formation of c-kitG20T, c-mycTGA-de and c-myc1245 G-quadruplex were evidenced by ^1^H-NMR (see Figure S30-S32) and CD spectra (Figure S33) and the CE of MACP6 has been enhanced with increasing G-quadruplex (see Figure S1-S3). Job plot analysis was carried out for MACP6/G4 stoichiometry determination^47^ Surprisingly, the peak value at 380 nm was observed ([MACP6]/([MACP6] + [G-quadruplex])) as 0.33 when G-quadruplex sequence is c-kitG20T or c-mycTGA-de (Fig. 5 and Figure S34-S35). The finding suggested that the binding stoichiometry between MACP6 and c-kitG20T or c-mycTGA-de is 1:2 (MACP6/G4). As we know, the 1:2 (MACP6/G4) complexation is unprecedented^48^,^49^. Differently, MACP6 and c-myc1245 adopted 1:1 binding mode (see Figure S36). Accordingly, it is noted that the different binding stoichiometry indicated the selectivity of G-quadruplex to MACP6, which give out one possible reason for the different stimuli response of MACP6/G4.

### Molecular modeling for binding mechanism

**Figure 6:**
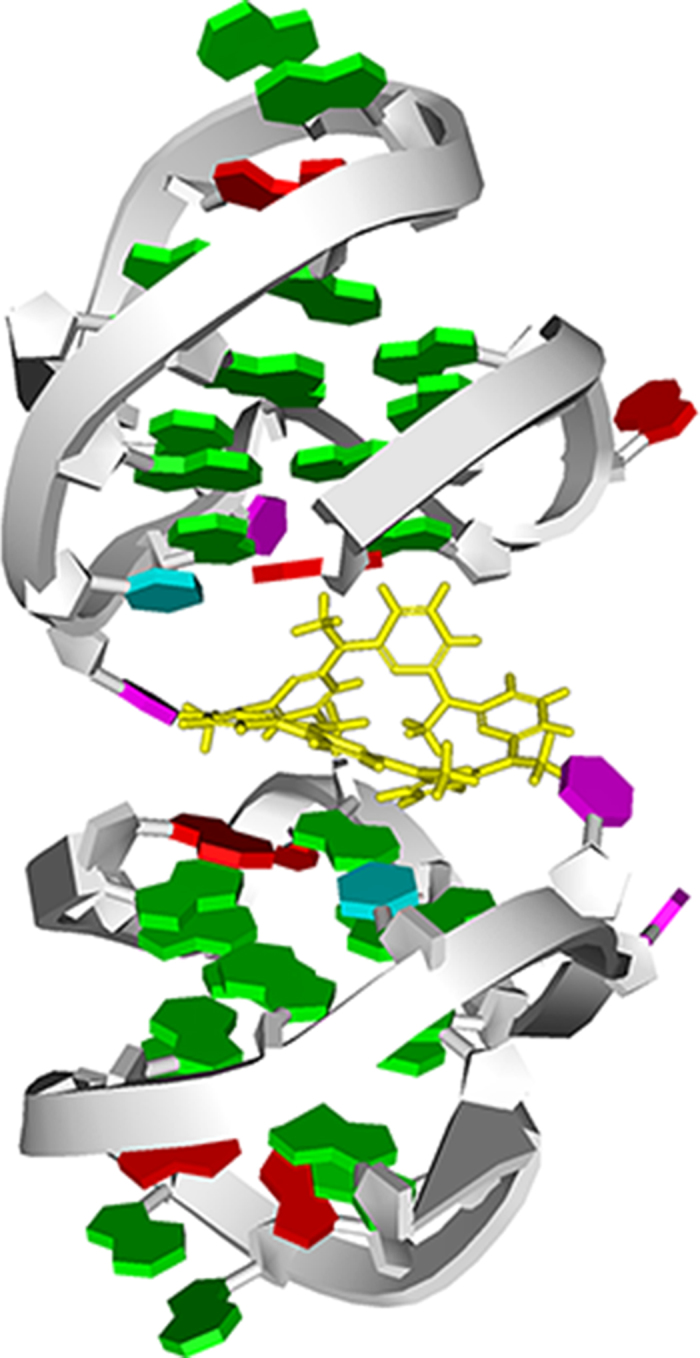
The plots of the docked structure of MACP6/c-kitG20T complex. MACP6 is colored yellow in the stick mode.

To get clear perception of the interaction between the c-kitG20T G-quadruplex and MACP6, molecular simulations were carried out. The molecular structure of MACP6 was first optimized using Discovery Studio 3.0 package and then docking it into the c-kitG20T G-quadruplex by Autodock 4.2^50^. The docking results showed that the MACP6 bonded to the site which was composed of two c-kitG20T G-quadruplex structures at the 5′-end top quartet (Fig. 6). When the temperature rises, the two c-kitG20T G-quadruplex structure begin to unfold from the 5′-end and MACP6 could be easily released. This binding mode may be favorable for the repeatability of the molecular switch.

### Binding affinity between MACP6 and G-quadruplexes

**Figure 7:**
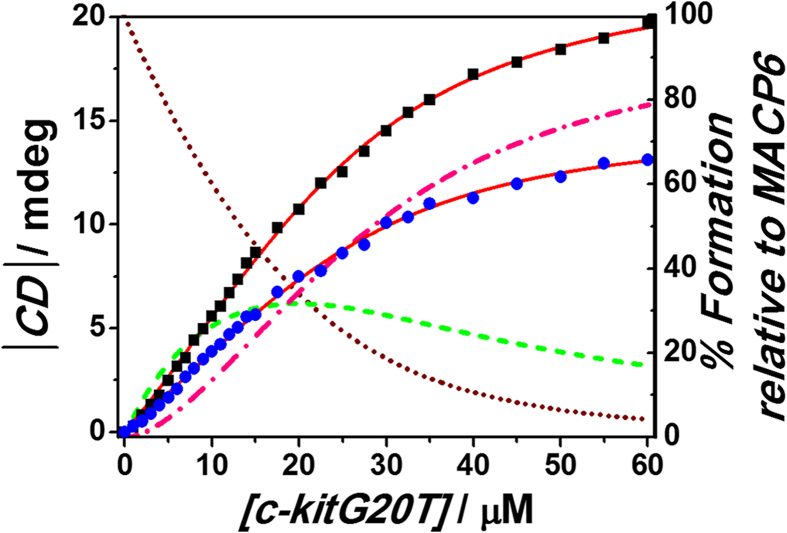
The CD titration isotherm of 10 μM MACP6 with the addition of c-kitG20T G-quadruplex in 17 mM phosphate buffer solution (5% DMSO) at 20 °C (⚬ or • indicate the change at 380 nm or 345 nm in CD spectra of MACP6, respectively). The CD spectra values from 320 nm to 430 nm were used for the calculation of *K*_*a1*_ ((9.4 ± 0.4) × 10^4^ M^−1^) and *K*_*a2*_ ((1.1 ± 0.07) × 10^5^ M^−1^) using the Hyperquad 2003 program^51^. The red lines show the least-square nonlinear fitting of the experimental data to the appropriate equations. The wine dot, green dash and pink dot dash lines show the calculated percentage of compound species including [MACP6], [MACP6•c-kitG20T] and [MACP6•(c-kitG20T)_2_] vs. the concentration of MACP6 at each additional c-kitG20T concentration.

The binding affinity of MACP6 for c-kitG20T was evaluated as associated constants (*K*_*a*_) as ((9.4 ± 0.4) × 10^4^ M^−1^) and ((1.1 ± 0.1) × 10^5^ M^−1^) with a good fit to a 1:1 and 1:2 binding profiles, respectively (Fig. 7). With analogue titration process, *K*_*a*_ as (5.1 ± 0.2) × 10^4^  M^−1^ and (2.8 ± 0.2) × 10^2^ M^−1^ between MACP6 and c-mycTGA-de was calculated for their 1:1 and 1:2 (MACP6:G4) complexation (see Figure S37). The finding suggested that even both G-quadruplex sequences (c-kitG20T and c-mycTGA-de) can bind to MACP6 with 2:1 (G4:MACP6) stoichiometry, different binding mode can then be predicted. For G-quadruplex c-kitG20T, similar constants were shown in 1:1 and 1:2 binding, implied that the first bonded G-quadruplex has little influence for the second G-quadruplex complexation, so both process contribute relatively equally to the induced chirality of MACP6. But in the case of c-mycTGA-de G-quadruplex, the *K*_*a*_ value of 1:2 (MACP6:G4) is much smaller (1/200) than the first one, indicated that formation of 1:1 complex are much more effective than 1:2 binding, with the propose that the 1:1 complex has significant contribution to the induced chirality compared to the 1:2 complex. Meanwhile, the CD titration curves could be fitted to give a *K*_*a*_ value as (1.3 ± 0.05) × 10^6^ M^−1^ with 1:1 (MACP6:G4) binding between c-myc1245 G-quadruplex and MACP6 (see Figure S38). Therefore, the different binding mode of MACP6 to the G-quadruplexes we studied indicated its selectivity to G-quadruplexes.

## Conclusions

In conclusion, we developed a switch based on G4/MACP6 controlled by temperature or pH value. MACP6 showed various thermal stimuli at the present of various G-quadruplexes. Discard molecular switching effects are of great interest in the design of environmentally responsive materials and molecular machines, the strategy we established has the potential to be used as the probe for specific G-quadruplex sequence recognition. We also expect the remarkable switchable operation of special G-quadruplex based supramolecular self-assembly architecture to open up new possibilities in the field of G-quadruplex recognition.

## Methods

### Sample preparation

All oligonucleotides (Table S1) were synthesized by Sangon Biotechnology (Shanghai, China) and purified by ultra-polyacrylamide gel electrophoresis (ULTRAPAGE) (purity 95%). Analytical grade DMSO, KH_2_PO_4_ K_2_HPO_4_ and ethylenediaminetetraacetic acid (EDTA) were purchased from Beijing Chem. Co. (China) and used without further purification. Ultrapure water prepared by Milli-Q Gradient ultrapure water system (Millipore) was used throughout the experiments. The solution of oligonucleotides were dissolved in 17 mM phosphate buffer solution (K_2_HPO_4_/KH_2_PO_4_, pH 7.40) and heated at 85 °C for 15 min, and then slowly cooled to room temperature. Methylazacalix[6]pyridine (MACP6) was synthesized according to the literature^52^ and thepurity was proved by element analysis^41^ MACP6 was dissolved in DMSO to obtain the stock solution. All the samples were prepared as shown above unless special instructions were given.

### Circular dichroism spectra measurements

Circular dichroism spectra were recorded from 230 nm to 450 nm with a JASCO J-810 spectropolarimeter equipped with a JASCO PTC-423 S temperature controller. Four scans were accumulated and averaged under 500 nm/min scanning speed, 2 nm bandwidth, 0.5 s response time and 0.2 nm data pitch. The solution has been stablized for 3 minutes at each temperature before collectting the data in variable temperature CD experiments. Purified nitrogen was applied to deoxygenate and kept the inert gas shielding during the experiments.

### NMR experiment

NMR experiments were performed on a Bruker AVANCE 600 spectrometer or a Bruker AVIII 500WB spectrometer equipped with a 5 mm BBI probe capable of delivering z-field gradients. The ^1^H-NMR spectra were recorded by the pulse program p3919gp that applied 3-9-19 pulses with gradients for water suppression, 1024 or 512 scans were acquired for each spectrum with a relaxation delay of 2 s. For each sample, trimethylsilyl propionate (TSP) was added as a reference of chemical shift.

### Job plot

CD spectroscopic Job plot was used to determine the binding stoichiometry. The total concentration of MACP6 and G-quadruplex (c-kitG20T, c-mycTGA-de or c-myc1245) were maintained at 12 μM, with the molar ratio of MACP6 and G-quadruplex as 1:0, 5:1, 4:1, 3:1, 2:1, 1.5:1, 1:1, 1:1.5, 1:2, 1:3, 1:4 and 1:5. The peak value shown in the plot corresponds to the stoichiometry which best describe the binding between MACP6 and G-quadruplex^47^.

### Nonlinear fitting and calculation

CD spectroscopic titration of MACP6 (the concentration was keep as 10 μM) with increasing G-quadruplex was carried out. The association constants *K*_*a*_ of the MACP6/G4 complexation were caculated with nonlinear fitting via the Hyperquad 2003 program^51^ based on the binding equilibrium profiles determined via Job plot.

### Molecular modeling

The 3D coordinates of c-kitG20T DNA G-quadruplex structure was retrieved from the RCSB Protein Data Bank. The structure of G-quadruplex were prepared for docking as described^50^. The molecular structure of MACP6 was optimized with MMFF force field using the Discovery Studio 3.5 (Accelrys Software Inc., San Diego). The molecular docking studies were carried out by using the Autodock 4.2 with Lamarckian genetic algorithm following the protocols developed for DNA G-quadruplex and ligand docking^50^. The figures were rendered using Discovery Studio 3.5.

## Author Contributions

A.-J.G. and H.-Y. G. designed the experiments and wrote the manuscript. M.-J. S. performed the experiment and wrote the supporting information; E.-X. Z. synthesized the MACP6; Q. L. performed the molecular modeling; J.-F. X. performed NMR experiment and commented on the manuscript; Q. L., H.-X. S., G.-Z. X. and L.-X. W. assisted with the analysis of chemical experiments; Y-L. T. and L.-J. X. advised on the project. All authors reviewed the manuscript.

## Additional Information

**How to cite this article**: Guan, A.-J. *et al.* G-quadruplex induced chirality of methylazacalix[6]pyridine via unprecedented binding stoichiometry: en route to multiplex controlled molecular switch. *Sci. Rep.* **5**, 10479; doi: 10.1038/srep10479 (2015).

## Supplementary Material

Supporting Information

## Figures and Tables

**Figure 1:**
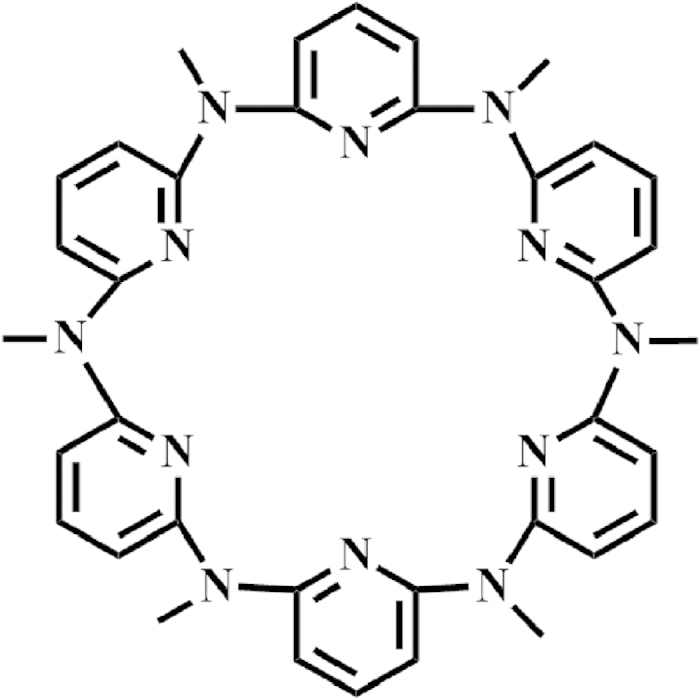
Structure of MACP6.
