# Supplementary material for: G-quadruplex induced chirality of methylazacalix[6]pyridine via unprecedented binding stoichiometry: en route to multiplex controlled molecular switch
Source: Sci Rep. 2015 May 20;5:10479. doi: 10.1038/srep10479 (PMC4438431; doi:10.1038/srep10479)
Supplement: Supporting Information [file srep10479-s1.pdf]

Electronic Supporting Information For:

G-quadraplex induced chirality of methylazacalix-[6]pyridine via unprecedented binding stoichiometry: en route to multiplex controlled molecular switch

Ai-Jiao Guan[1], Meng-Jie Shen [2], Jun-Feng Xiang[1], En-Xuan Zhang[1], Qian Li[1], Hong-Xia Sun[1], Li-Xia Wang[1], Guang-Zhi Xu[1], Ya-Lin Tang[1]*, Li-Jin Xu[2]* & Han-Yuan Gong[3]*

Contents

1. Materials and methods..3

1.1 Sample preparation 3

1.2 Circular dichroism spectra measurements..3

1.3 NMR experiment. 4

1.4 Job plot 4

1.5 Nonlinear fitting and calculation 4

1.6 Molecular modeling...5

2. Tables and figures 5

3. References 27

1. Materials and methods

1.1 Sample preparation

All oligonucleotides (Table S1) were synthesized by Sangon Biotechnology (Shanghai, China) and purified by ultra-polyacrylamide gel electrophoresis (ULTRAPAGE) (purity 95%). Analytical grade DMSO, KH_2PO_4, K_2HPO_4 and ethylenediaminetetraacetic acid (EDTA) were purchased from Beijing Chem. Co. (China) and used without further purification. Ultrapure water prepared by Milli-Q Gradient ultrapure water system (Millipore) was used throughout the experiments. The solution of oligonucleotides were dissolved in 17 mM phosphate buffer solution (K_2HPO_4/KH_2PO_4, pH 7.40) and heated at 85 ℃ for 15 min, and then slowly cooled to room temperature. The concentration of each DNA sample was determined via absorbance extinction coefficient value of 260 nm at 25 ℃. Methylazacalix[6]pyridine (MACP6) was synthesized according to the literature[1] and the purity was proved by element analysis.[2] MACP6 was dissolved in DMSO to obtain the stock solution. All the samples were prepared as shown above unless special instructions were given.

1.2 Circular dichroism spectra measurements

Circular dichroism spectra were recorded from 200 nm to 450 nm with a JASCO J-810 or J-815 spectropolarimeter equipped with a JASCO PTC-423S temperature controller. Four scans were accumulated and averaged under 500 nm/min scanning speed, 2 nm bandwidth, 0.5 s response time and 0.2 nm data pitch. The solution have been stablized for 3 minutes at each temperature before collectting the data in variable temperature CD experiments. Purified nitrogen was applied to deoxygenate and kept the inert gas shielding during the experiments.

1.3 NMR experiment.

The NMR experiments were either performed on a Bruker AVIII 500WB spectrometer (FigureS9-S10, S14-S15 and S17-S18) or a Bruker AVANCE 600 spectrometer (Figure S30-S32). Both of them equipped with a 5 mm BBI probe capable of delivering z-field gradients. The ^{1}H-NMR spectra were recorded by the pulse program p3919gp that applied 3-9-19 pulses with gradients for water suppression, 1024 or 512 scans were acquired for each spectrum with a relaxation delay of 2 s. The temperature of sample was stabilized for 10 minutes before collecting spectra after a targeted tempetature in variable temperature NMR experiments. For each sample, trimethylsilyl propionate (TSP) was added as a reference of chemical shift

1.4 Job plot

CD spectroscopic Job plot was used to determine the binding stoichiometry. The total concentration of MACP6 and G-quadruplex (c-kitG20T, c-mycTGA-de or c-myc1245) were maintained at 12 μM, with the molar ratio of MACP6 and G-quadruplex as 1:0, 5:1, 4:1, 3:1, 2:1, 1.5:1, 1:1, 1:1.5, 1:2, 1:3, 1:4 and 1:5. The peak value shown in the plot corresponds to the stoichiometry which best describe the binding between MACP6 and G-quadruplex.[3]

1.5 Nonlinear fitting and calculation

CD spectroscopic titration of MACP6 (the concentration was keep as 10 μM) with increasing G-quadruplex was carried out. The association constants K_a of the MACP6/G4 complexation were caculated with nonlinear fitting via the Hyperquad 2003 program[4] based on the binding equilibrium profiles determined via Job plot.

1.6 Molecular modeling

The 3D coordinates of c-kitG20T DNA G-quadruplex structure was retrieved from the RCSB Protein Data Bank. The G-quadruplex structures were prepared for docking as described.[5] The molecular structure of MACP6 was optimized with MMFF force field using the Discovery Studio 3.5 (Accelrys Software Inc., San Diego). The molecular docking studies were carried out by using the Autodock 4.2 with Lamarckian genetic algorithm following the protocols developed for DNA G-quadruplex and ligand docking.[5] The figures were rendered using Discovery Studio 3.5.

2. Tables and figures

Table S1. DNA oligomers sequence

Name	*Sequence*
c-kitG20T	5‘-AGGGAGGGCGCTGGGAGGATGG-3‘
c-mycTGA-de	5‘-GGGTGGGGAGGGTGGGGAA-3‘
c-myc1245	5‘-TGAGGGTGGGTTTTTAGGGTGGGGAA-3‘

Table S2. The melting temperature of each DNA G-quadruplex

G-quadruplex	*Melting temperature / ℃*
c-kitG20T	46
c-mycTGA-de	75
c-myc1245	66

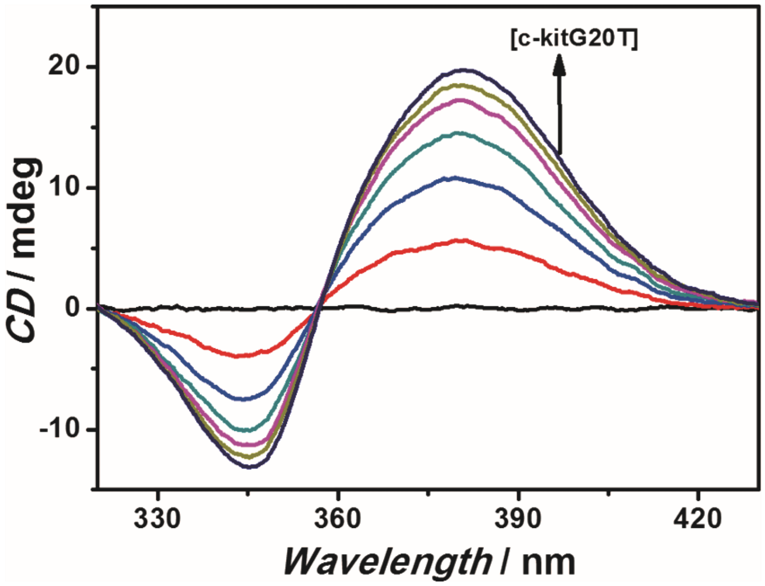

Figure S1. The CD spectra of 10 μM MACP6 with increasing c-kitG20T G-quadruplex (from 0 to 60 μM) in 17 mM phosphate buffer solution (5% DMSO) at 20 ℃.

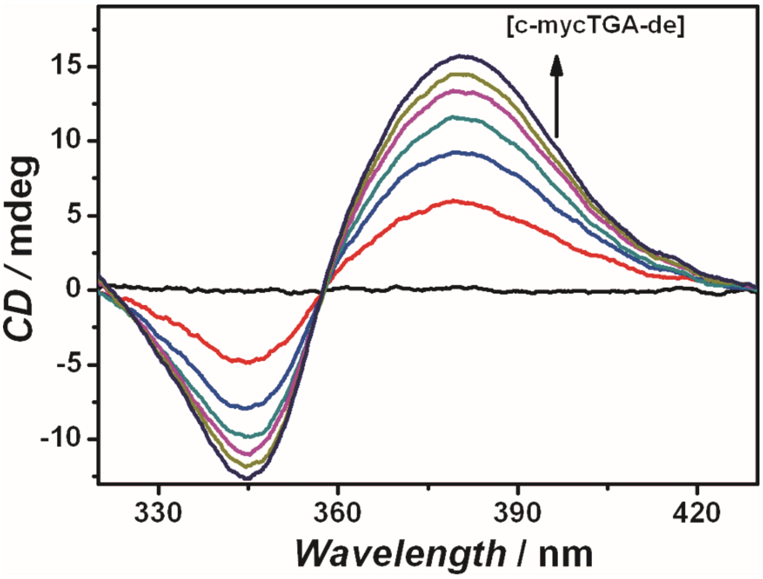

Figure S2. The CD spectra of 10 μM MACP6 with increasing c-mycTGA-de G-quadruplex (from 0 to 60 μM) in 17 mM phosphate buffer solution (5% DMSO) at 20 ℃.

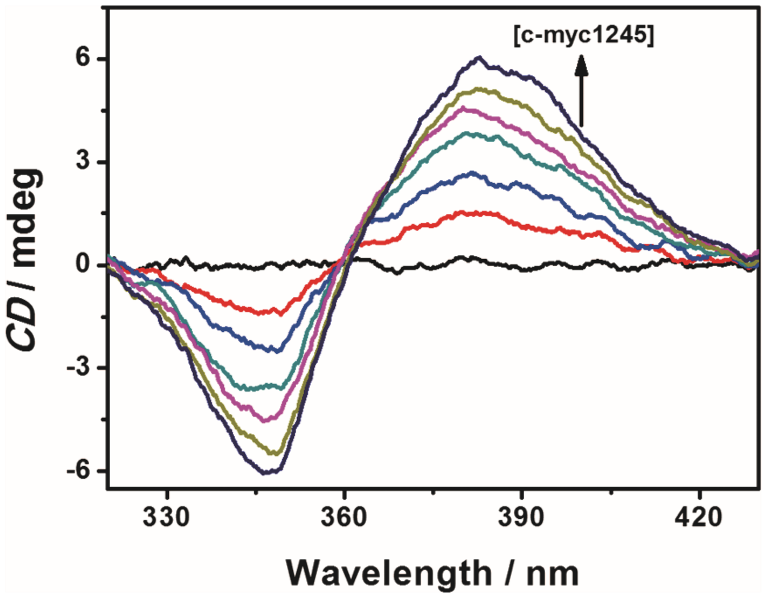

Figure S3. The CD spectra of 10 μM MACP6 with increasing c-myc1245 G-quadruplex (from 0 to 60 μM) in 17 mM phosphate buffer solution (5% DMSO) at 20 ℃.

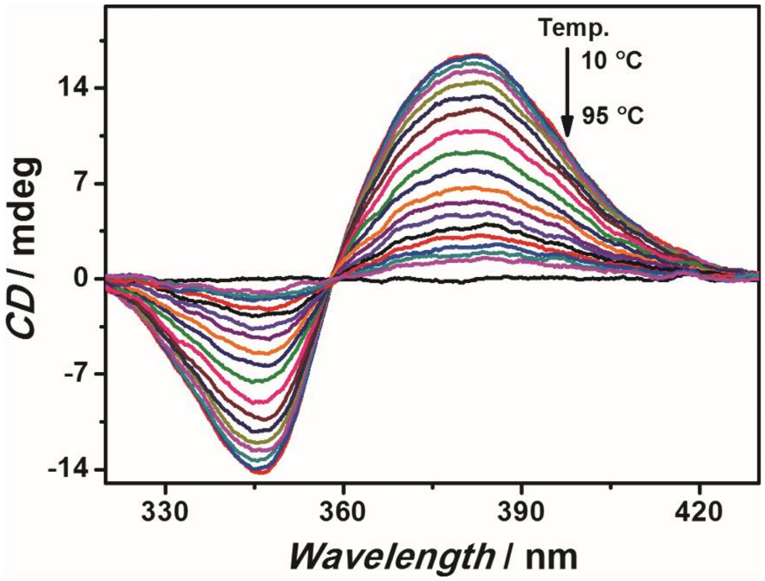

Figure S4. The CD spectra of the mixture containing MACP6 (10 μM) and c-mycTGA-de (20 μM) corresponding to different temperatures (from 10 ℃ to 95 ℃) in 17 mM phosphate buffer solution (5% DMSO). At every temperature, the solution have been left to staid 3 minutes to stablize the equilibrium.

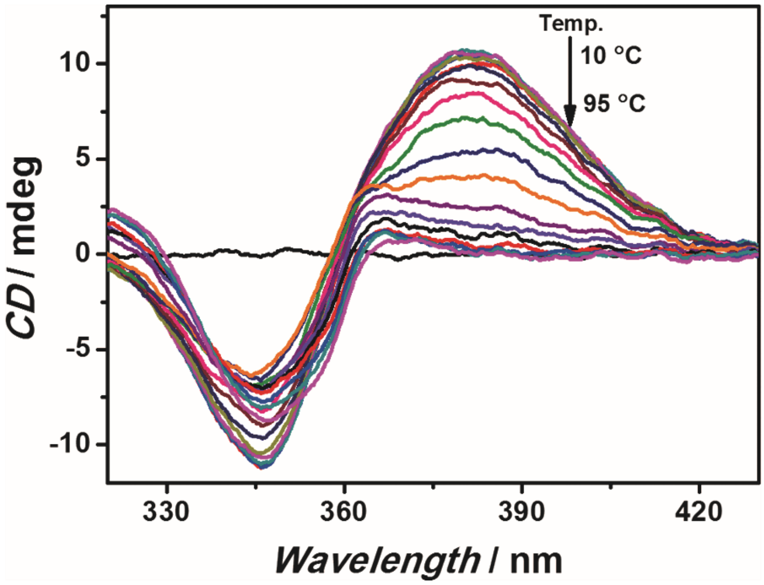

Figure S5. The CD spectra of the mixture containing MACP6 (10 μM) and c-myc1245 (20 μM) corresponding to different temperatures (from 10 ℃ to 95 ℃) in 17 mM phosphate buffer solution (5% DMSO). At every temperature, the solution have been left to staid 3 minutes to stablize the equilibrium.

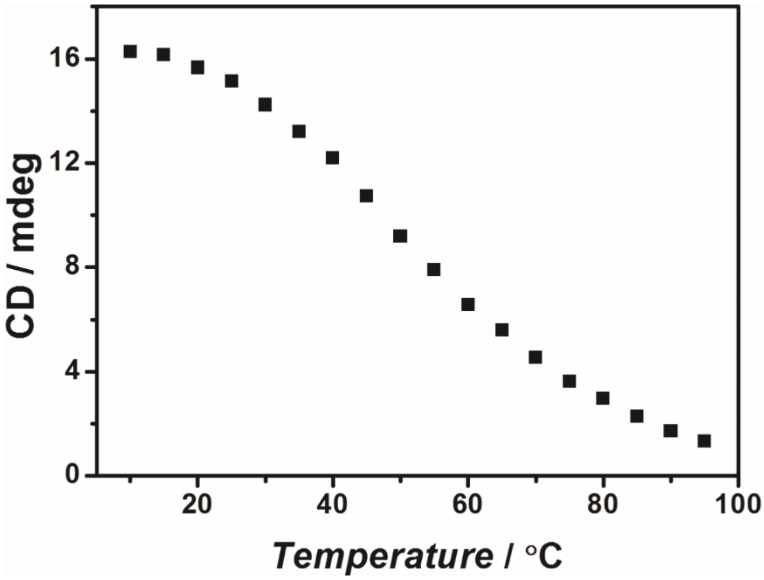

Figure S6. The CD intensity (380 nm) of the mixture containing MACP6 (10 μM) and c-mycTGA-de (20 μM) change with increasing temperature (from 10 ℃ to 95 ℃) in 17 mM phosphate buffer solution (5% DMSO) at pH 7.40.

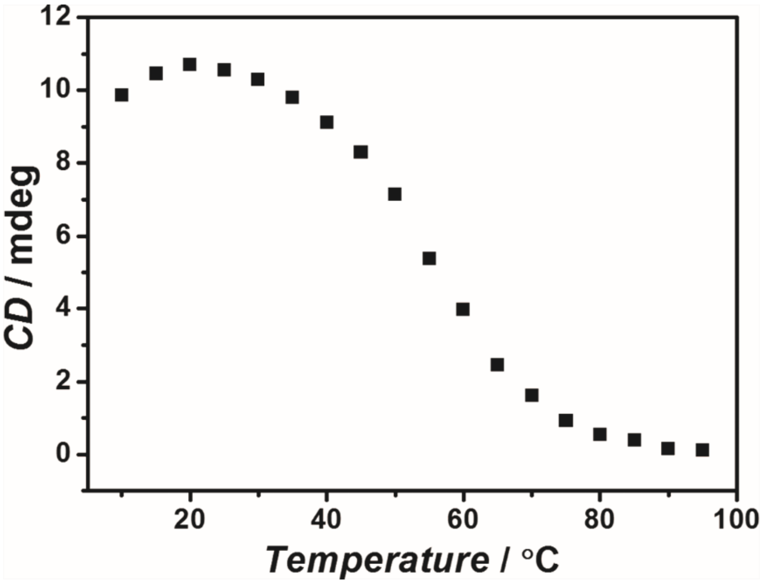

Figure S7. The CD intensity (380 nm) of the mixture containing MACP6 (10 μM) and c-myc1245 (20 μM) change with increasing temperature (from 10 ℃ to 95 ℃) in 17 mM phosphate buffer solution (5% DMSO) at pH 7.40.

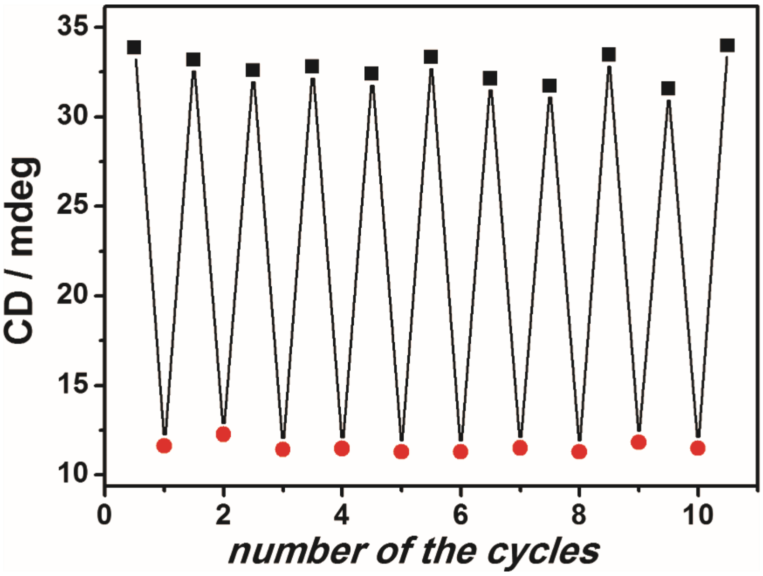

Figure S8. The CD intensity (265 nm) of the c-kitG20T (5 μM) in 17 mM phosphate buffer solution at 20 ℃ (represented as"■") and 60 ℃ (represented as"●") were used to monitor the activity of the c-kitG20T G-quadruplex during the cycles of temperature.

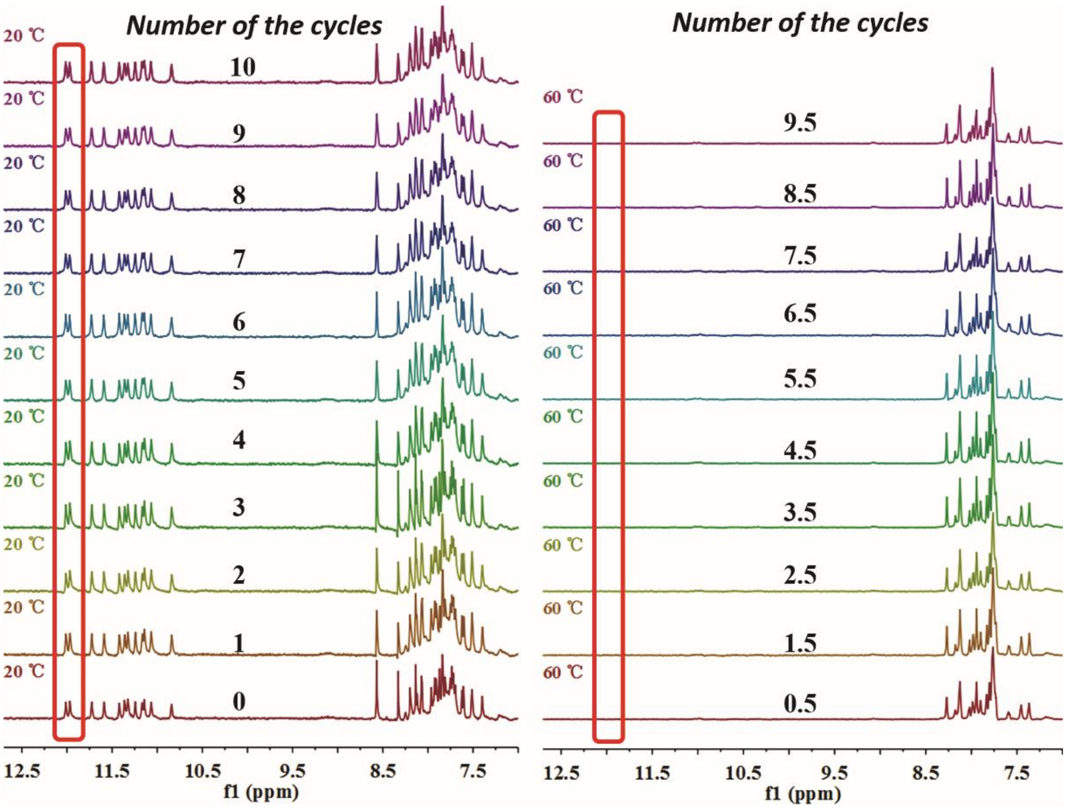

Figure S9. The ^{1}H-NMR spectra of c-kitG20T G-quadruplex under temperature cycling from 20 ℃ to 60 ℃. The integral area of the peak in the red frame was used to monitor the formation and dissociation of the c-kitG20T G-quadruplex as show in figure S10.

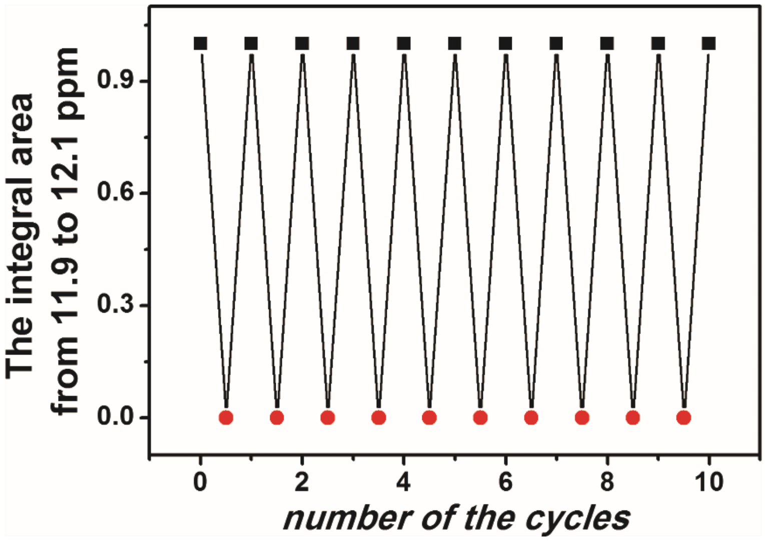

Figure S10. The integral area of the peak from 11.9 to 12.1 ppm at 20 ℃ (represented as"■") and 60 ℃ (represented as"●") was used to monitor the formation and dissociation of the c-kitG20T G-quadruplex.

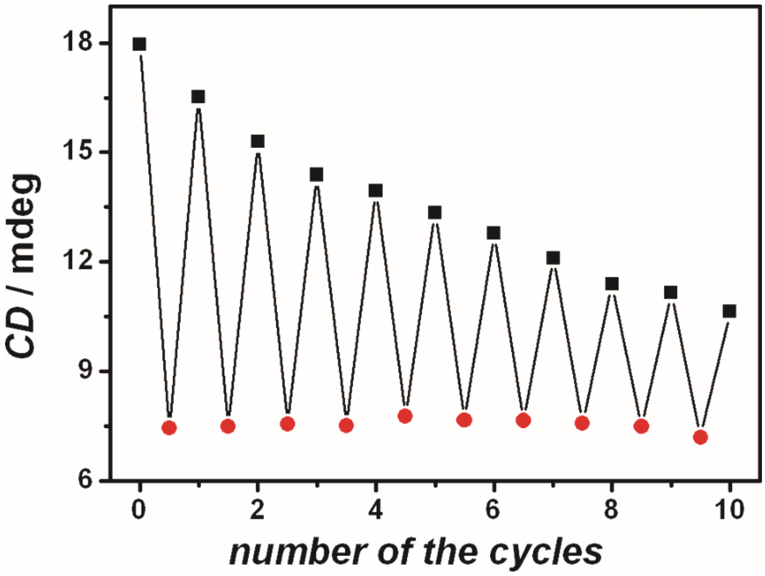

Figure S11. The CD intensity (380 nm) of the mixture containing MACP6 (10 μM) and c-mycTGA-de (20 μM) in 17 mM phosphate buffer solution (5% DMSO) at 25 ℃ (represented as"■") and 65 ℃ (represented as"●") were used to monitor the switching "on" and "off" of the molecular switch.

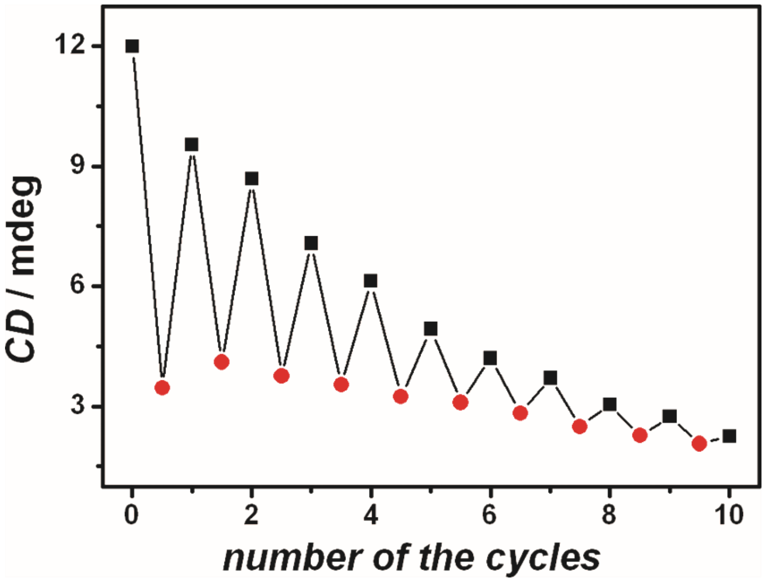

Figure S12. The CD intensity (380 nm) of the mixture containing MACP6 (10 μM) and c-myc1245 (20 μM) in 17 mM phosphate buffer solution (5% DMSO) at 25 ℃ (represented as"■") and 65 ℃ (represented as"●") were used to monitor the switching "on" and "off" of the molecular switch.

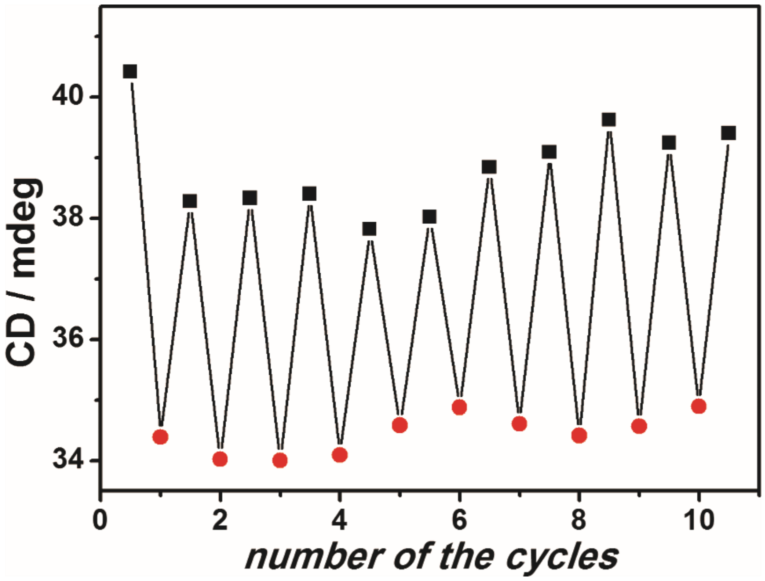

Figure S13. The CD intensity (263 nm) of the c-mycTGA-de (5 μM) in 17 mM phosphate buffer solution at 25 ℃ (represented as"■") and 65 ℃ (represented as"●") were used to monitor the activity of the c-mycTGA-de G-quadruplex during the cycles of temperature.

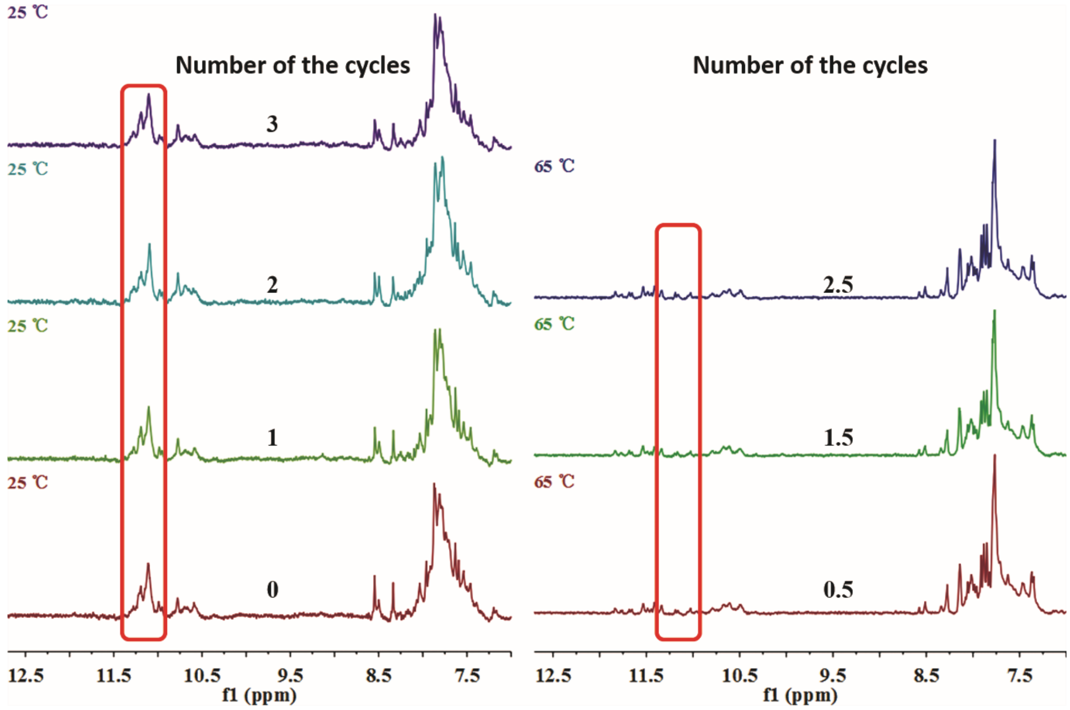

Figure S14. The ^{1}H-NMR spectra of c-mycTGA-de G-quadruplex under temperature cycling from 25 ℃ to 65 ℃. The integral area of the peak in the red frame was used to monitor the formation and dissociation of the c-mycTGA-de G-quadruplex as show in figure S15.

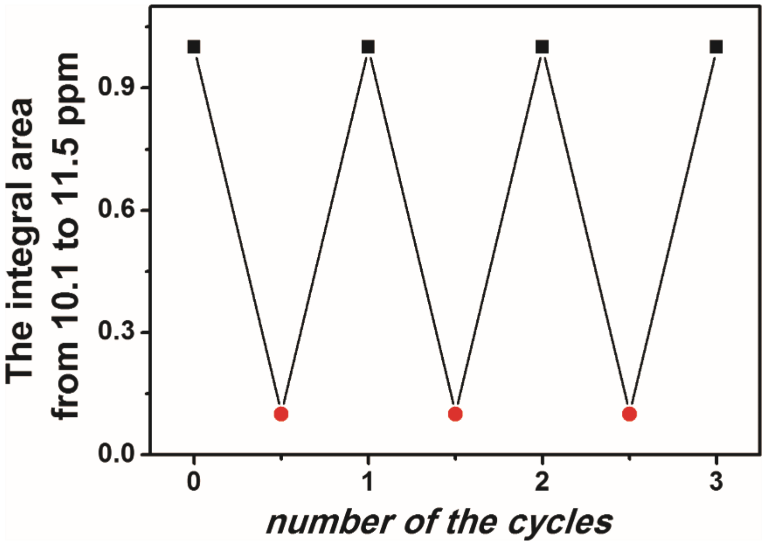

Figure S15. The integral area of the peak from 10.1 to 11.5 ppm at 25 ℃ (represented as"■") and 65 ℃ (represented as"●") was used to monitor the formation and dissociation of the c-mycTGA-de G-quadruplex.

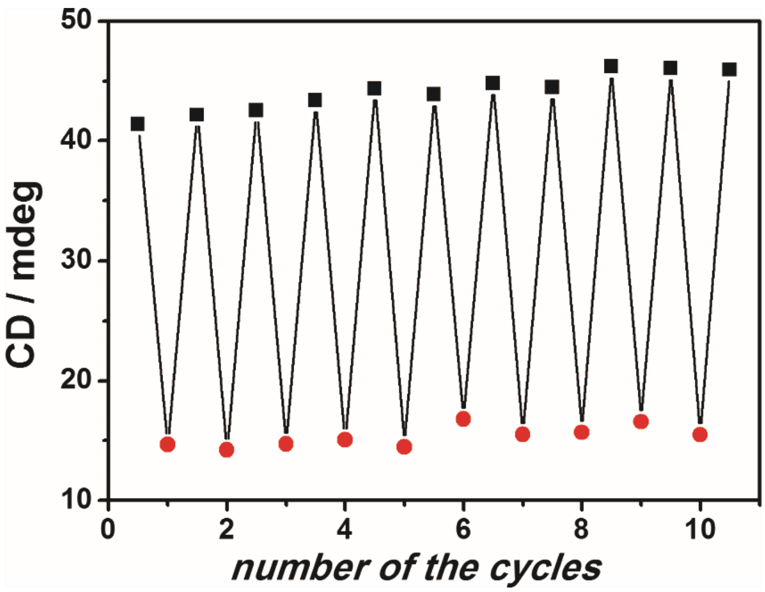

Figure S16. The CD intensity (265 nm) of the c-myc1245 (5 μM) in 17 mM phosphate buffer solution at 25 ℃ (represented as"■") and 65 ℃ (represented as"●") were used to monitor the activity of the c-myc1245 G-quadruplex during the cycles of temperature.

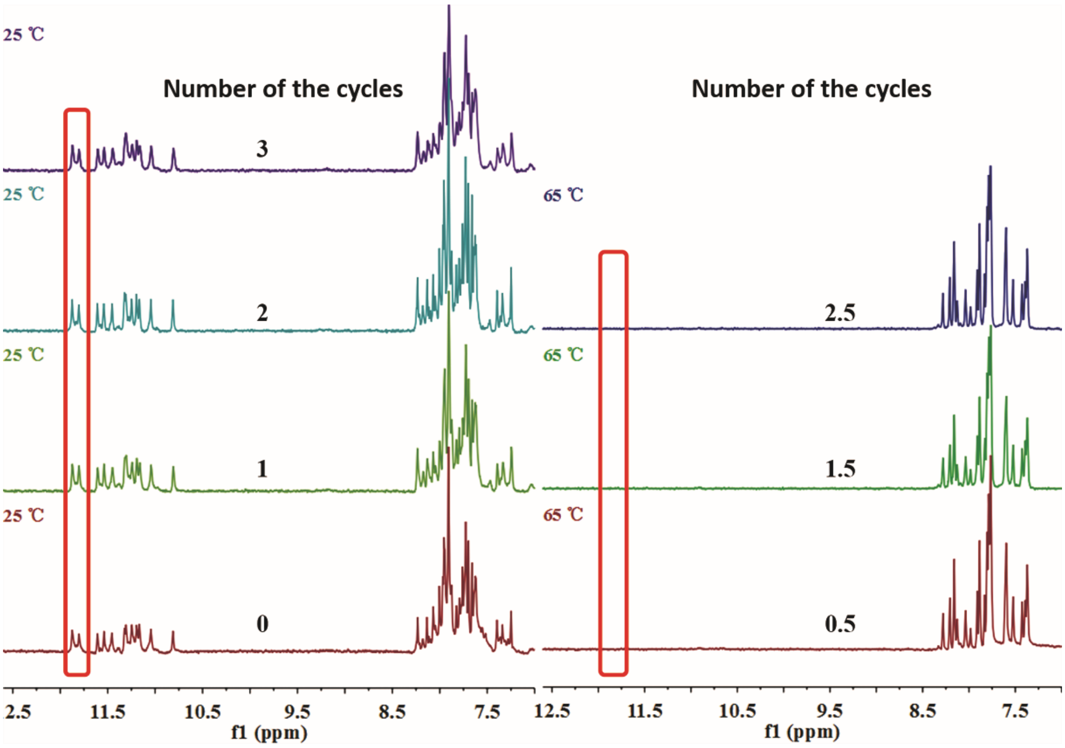

Figure S17. The ^{1}H-NMR spectra of c-myc1245 G-quadruplex under temperature cycling from 25 ℃ to 65 ℃. The integral area of the peak in the

red frame was used to monitor the formation and dissociation of the c-myc1245 G-quadruplex as show in figure S18.

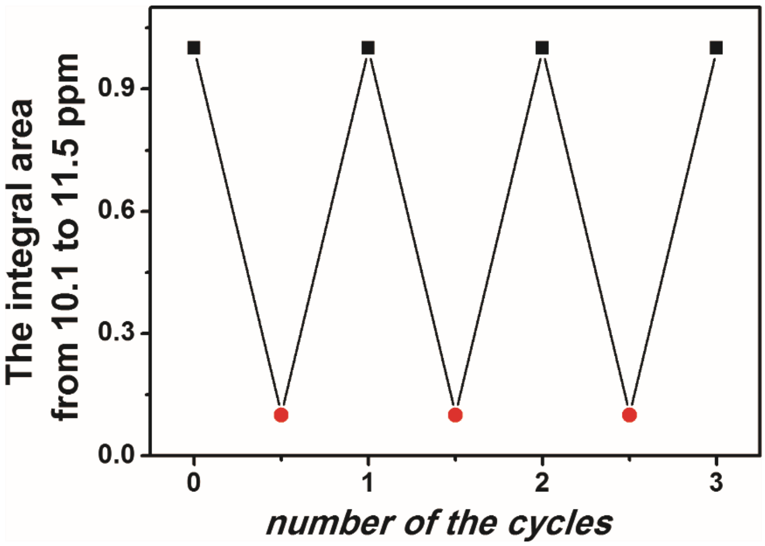

Figure S18. The integral area of the peak from 11.7 to 12.0 ppm at 25 ℃ (represented as“■”) and 65 ℃ (represented as“●”) was used to monitor the formation and dissociation of the c-myc1245 G-quadruplex.

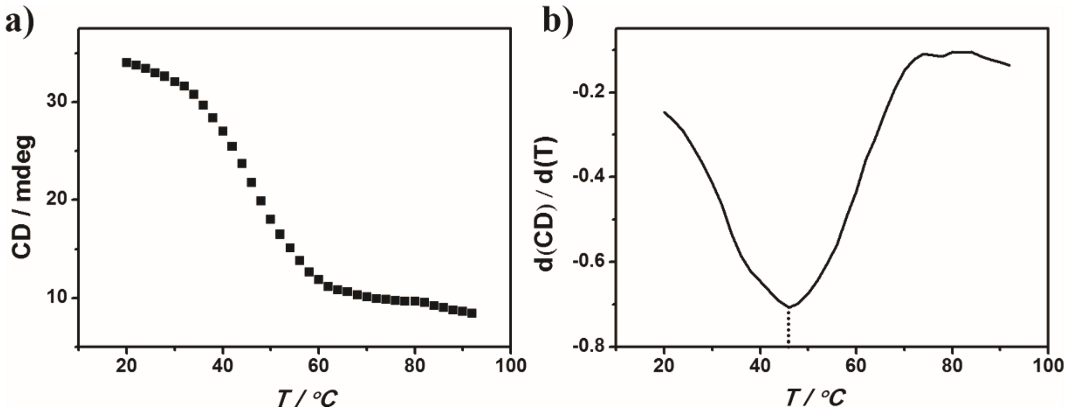

Figure S19. a) The CD intensity (265 nm) of the c-kitG20T G-quadruplex (5 μM) with temperature increasing from 20 ℃ to 92 ℃ in 17 mM phosphate buffer solution. b) The first-order derivative of CD intensity (265 nm) versus temperature, the abscissa of the lowest is 46 ℃, which corresponding to the denature temperature of the c-kitG20T G-quadruplex.

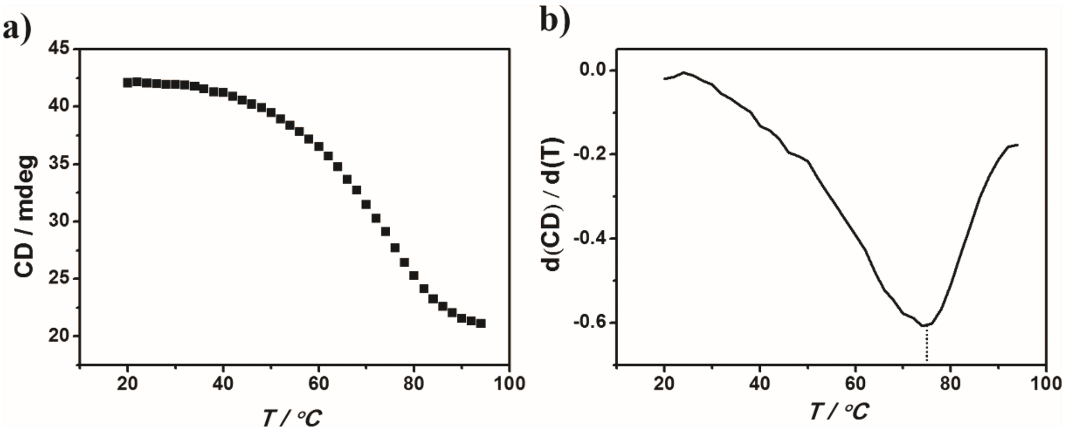

Figure S20. a) The CD intensity (263 nm) of the c-mycTGA-de G-quadruplex (5 μM) with temperature increasing from 20 ℃ to 94 ℃ in 17 mM phosphate buffer solution. b) The first-order derivative of CD intensity (263 nm) versus temperature, the abscissa of the lowest is 75 ℃, which corresponding to the denature temperature of the c-mycTGA-de G-quadruplex.

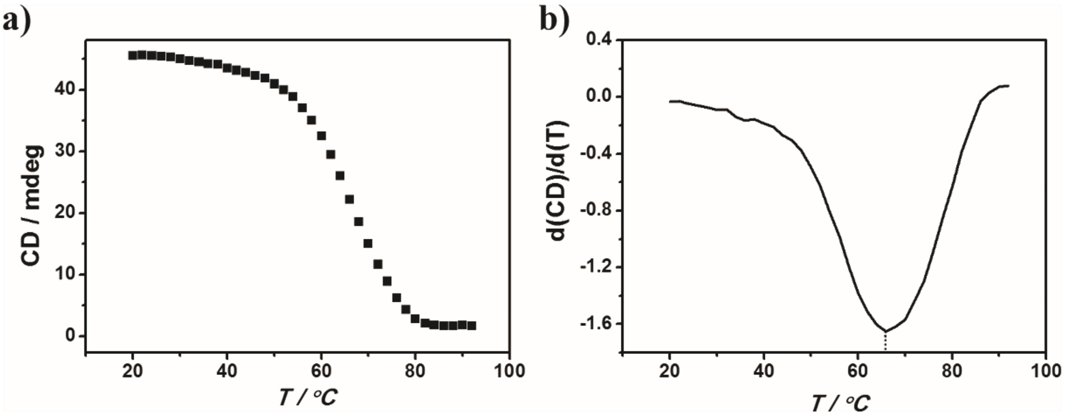

Figure S21. a) The CD intensity (265 nm) of the c-myc1245 G-quadruplex (5 μM) with temperature increasing from 20 ℃ to 92 ℃ in 17 mM phosphate buffer solution. b) The first-order derivative of CD intensity (265 nm) versus temperature, the abscissa of the lowest is 66 ℃, which corresponding to the denature temperature of the c-myc1245 G-quadruplex.

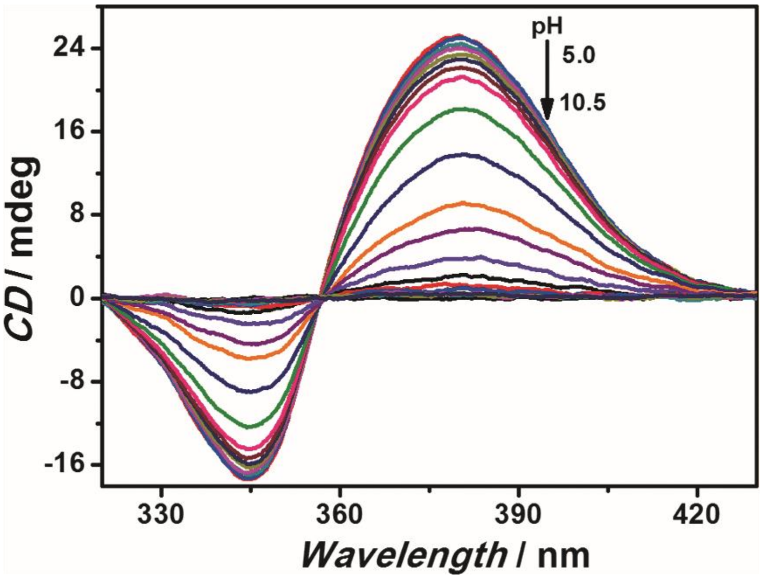

Figure S22. The CD spectra of the mixture containing MACP6 (10 μM) and c-kitG20T (20 μM) corresponding to different pH (from 5.0 to 10.5) in 17 mM phosphate buffer solution (5% DMSO).

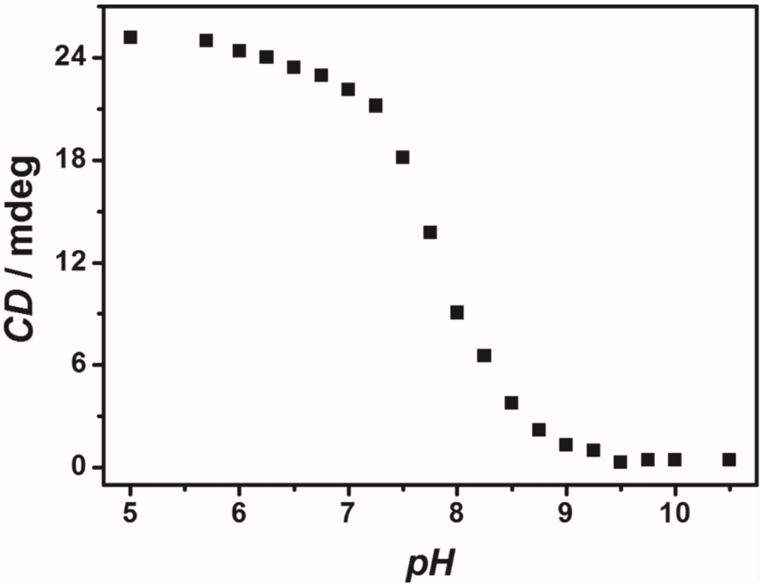

Figure S23. The CD intensity (380 nm) of the mixture containing MACP6 (10 μM) and c-kitG20T (20 μM) change with increasing pH value (from pH 5.0 to pH 10.5） in 17 mM phosphate buffer solution (5% DMSO).

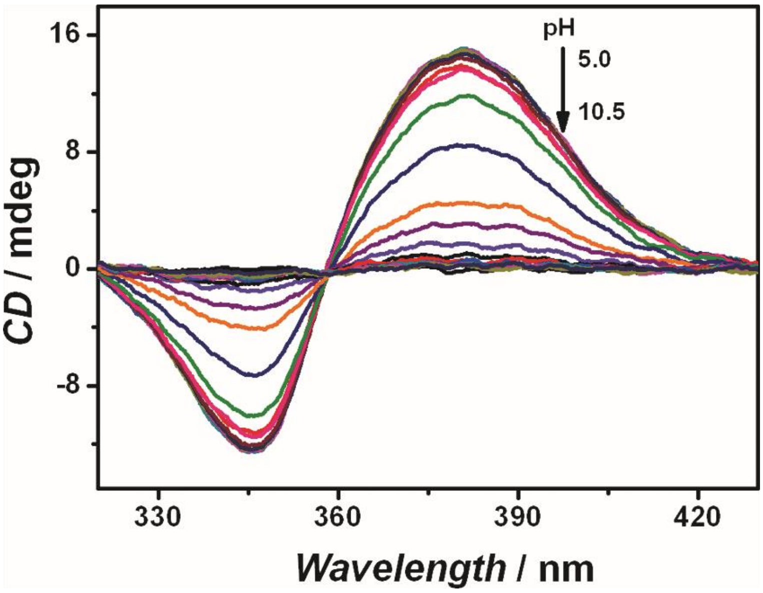

Figure S24. The CD spectra of the mixture containing MACP6 (10 μM) and c-mycTGA-de (20 μM) corresponding to different pH (from 5.0 to 10.5) in 17 mM phosphate buffer solution (5% DMSO).

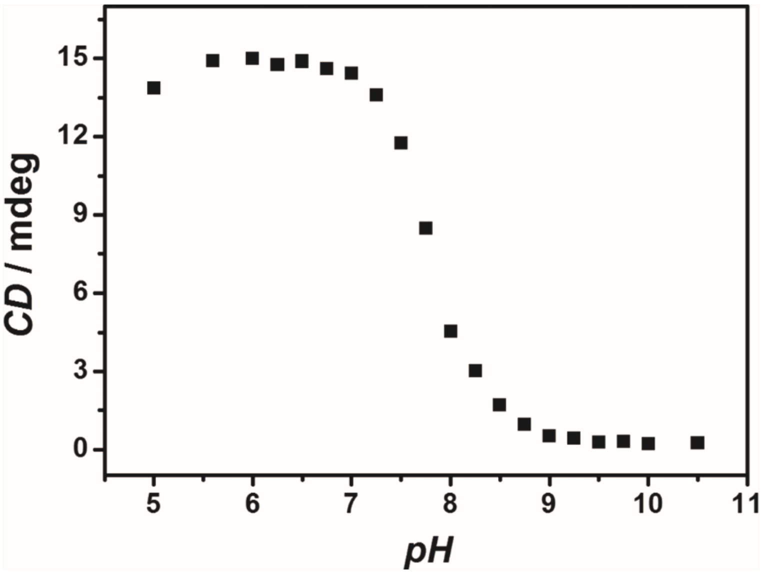

Figure S25. The CD intensity (380 nm) of the mixture containing MACP6 (10 μM) and c-mycTGA-de (20 μM) change with increasing pH value (from pH 5.0 to pH 10.5) in 17 mM phosphate buffer solution (5% DMSO).

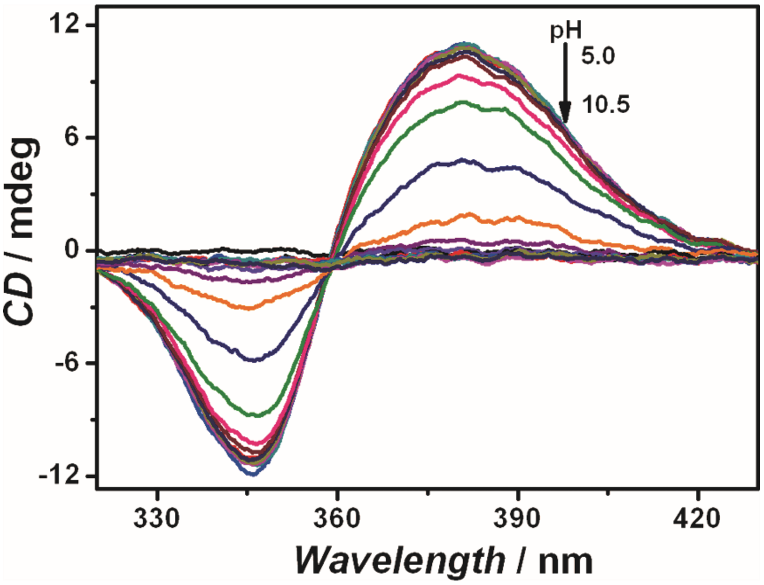

Figure S26. The CD spectra of the mixture containing MACP6 (10 μM) and c-myc1245 (20 μM) corresponding to different (pH from 5.0 to 10.5) in 17 mM phosphate buffer solution (5% DMSO).

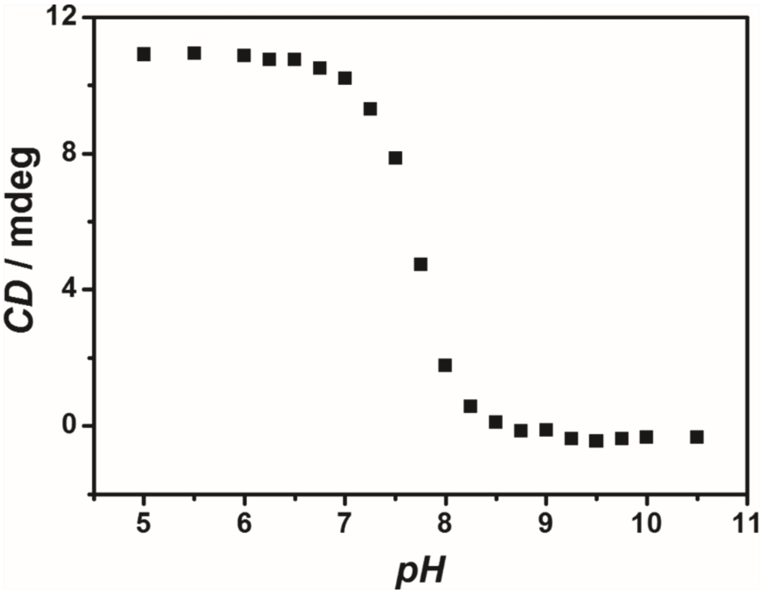

Figure S27. The CD intensity (380 nm) of the mixture containing MACP6 (10 μM) and c-myc1245 (20 μM) change with increasing pH value (from pH 5.0 to pH 10.5) in 17 mM phosphate buffer solution (5% DMSO).

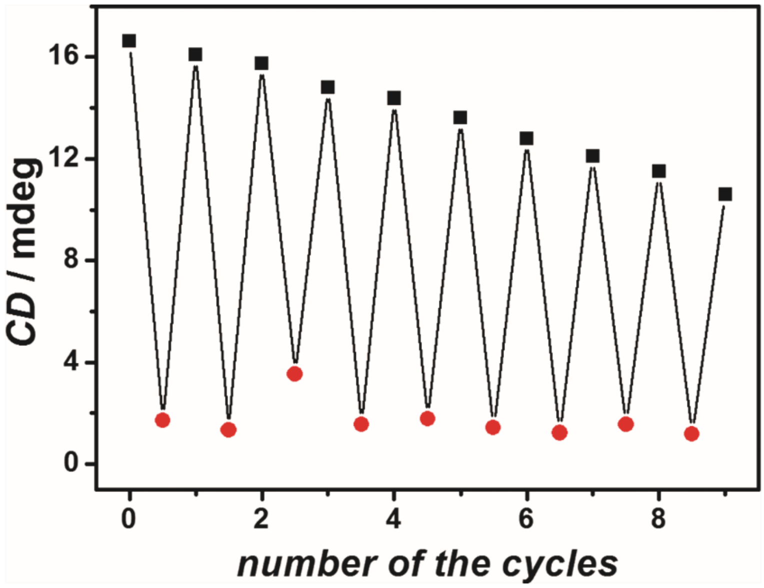

Figure S28. The CD intensity (380 nm) of the mixture containing MACP6 (10 μM) and c-mycTGA-de (20 μM) in 17 mM phosphate buffer solution (5% DMSO) at pH 6.0 (represented as"■") and pH 8.5 (represented as"●") were used to monitor the switching "on" and "off" of the molecular switch.

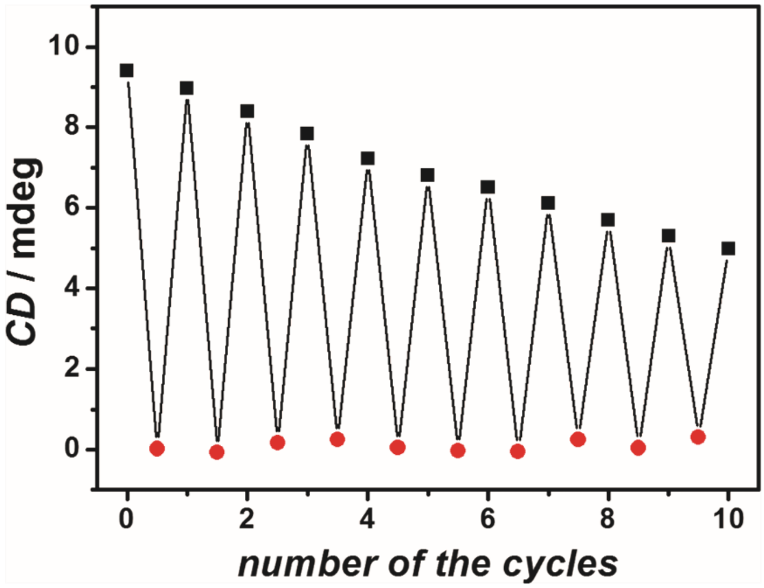

Figure S29. The CD intensity (380 nm) of the mixture containing MACP6 (10 μM) and c-myc1245 (20 μM) in 17 mM phosphate buffer solution (5% DMSO) at pH 6.0 (represented as"■") and pH 8.5 (represented as"●") were used to monitor the switching "on" and "off" of the molecular switch.

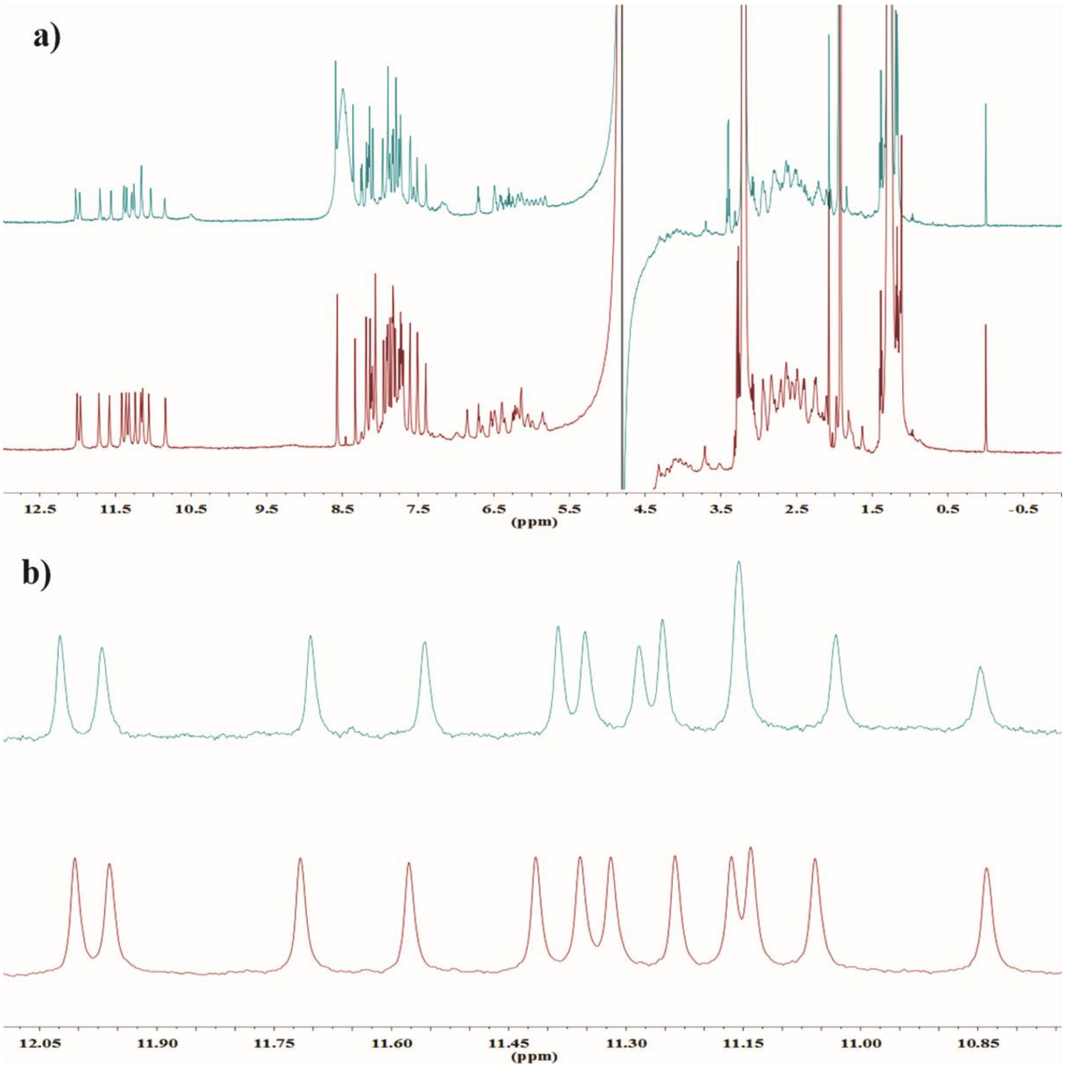

Figure S30. a) The full view and b) the expanded view of ^{1}H-NMR spectrum of c-kitG20T corresponding to pH 6.0 (green lines) and pH 8.5 (red lines), respectively.

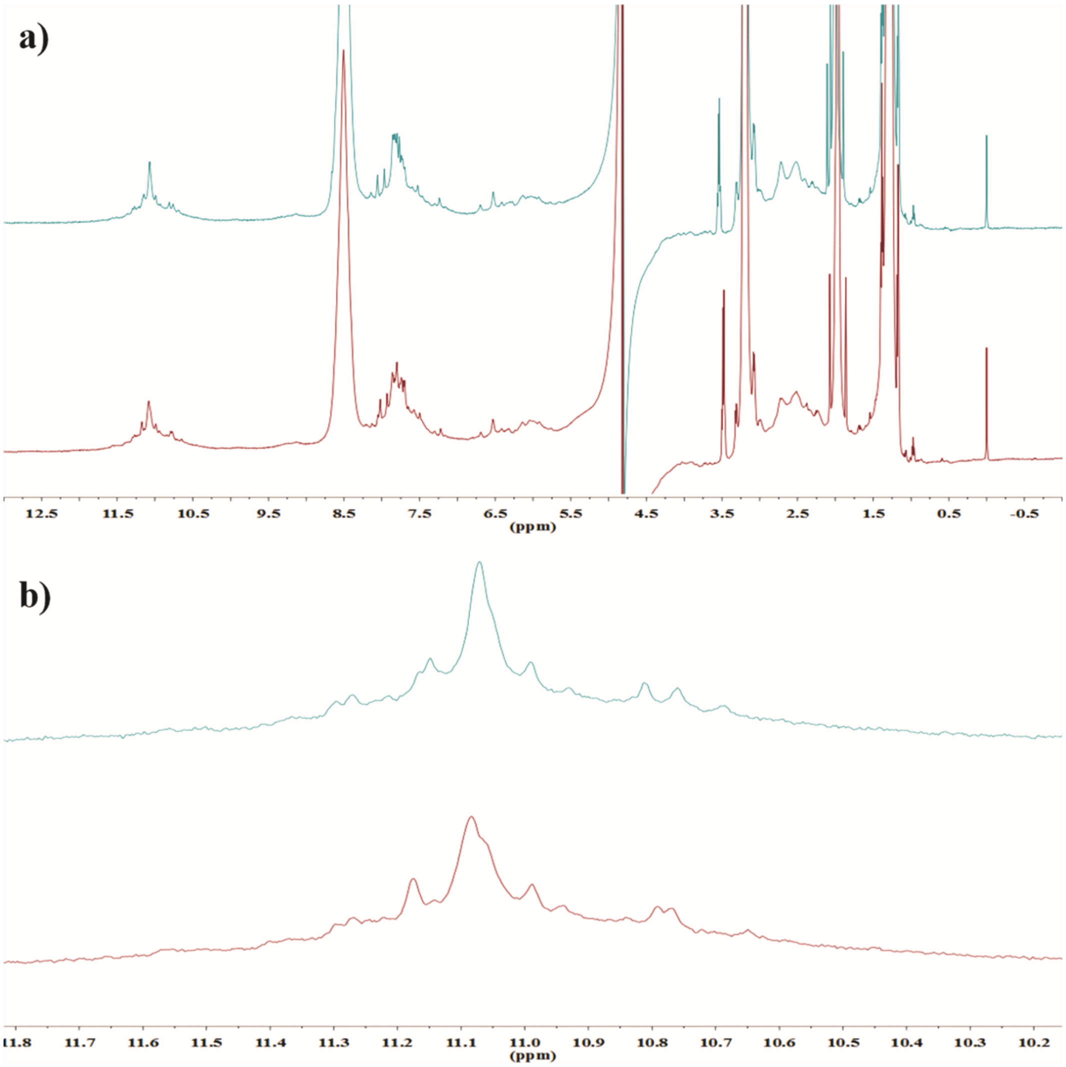

Figure S31. a) The full view and b) the expanded view of ^{1}H-NMR spectrum of c-mycTGA-de corresponding to pH 6.0 (green lines) and pH 8.5 (red lines), respectively.

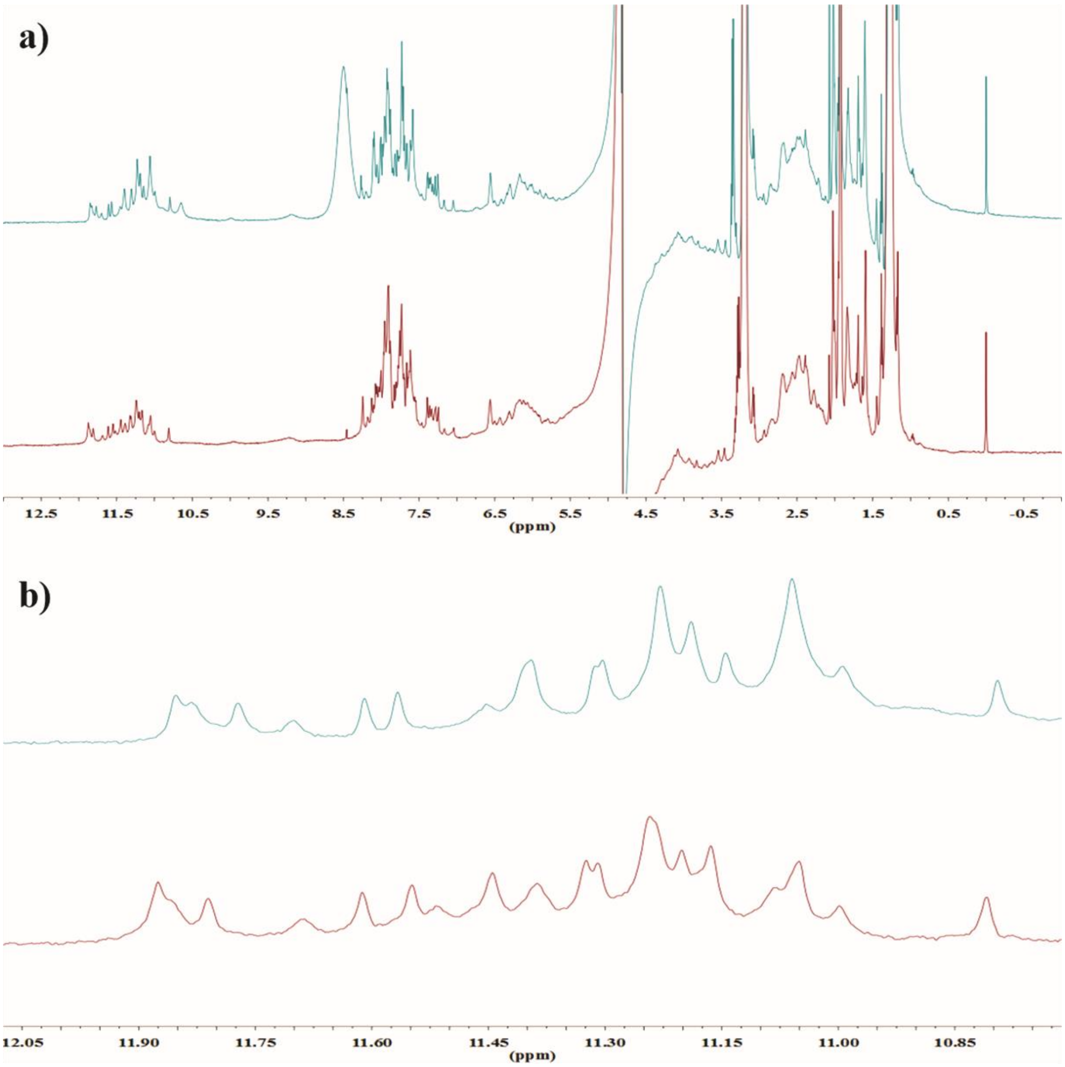

Figure S32. a) The full view and b) the expanded view of ^{1}H-NMR spectrum of c-myc1245 corresponding to pH 6.0 (green lines) and pH 8.5 (red lines), respectively.

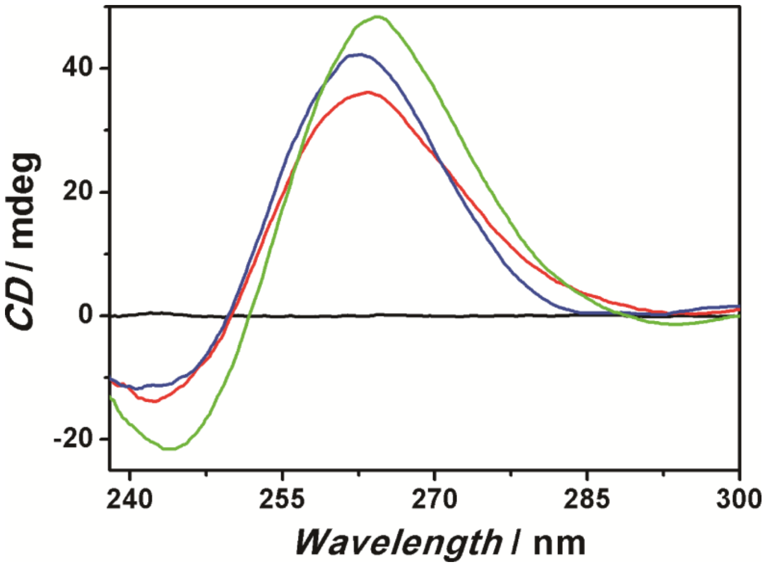

Figure S33. The CD spectra of the c-kitG20T (red line), c-mycTGA-de (blue line) and c-myc1245 (green line) G-quadruplexes (6 μM) in 17 mM phosphate buffer solution (5% DMSO) at 20 ℃..

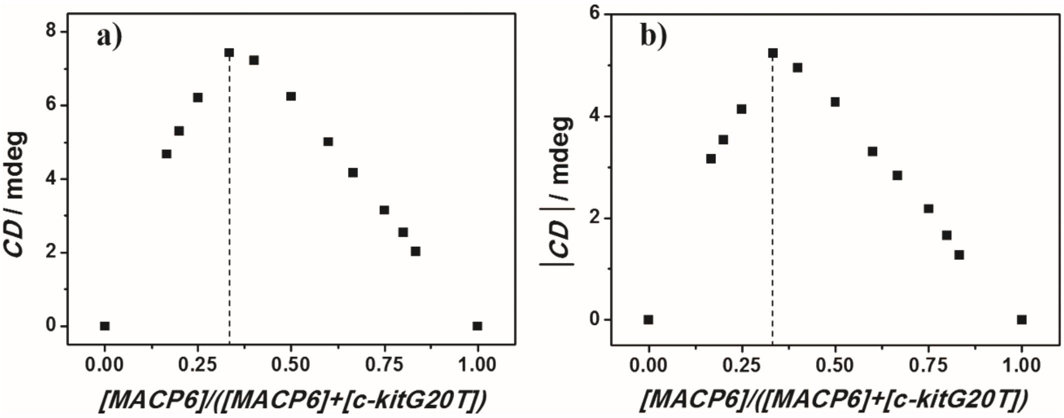

Figure S34. The CD Job plot corresponding to the binding between MACP6 and c-kitG20T in 17 mM phosphate buffer solution (5% DMSO) at 20 ℃. [MACP6] + [c-kitG20T] = 12 μM. At two wavelength (380 nm (a) and 345 nm (b)), the maximum value of y (defined as the CD intensity value of the MACP6/c-kitG20T complex) was found at 0.33, a finding consistent with a 1:2 (MACP6:c-kitG20T) binding stoichiometry.

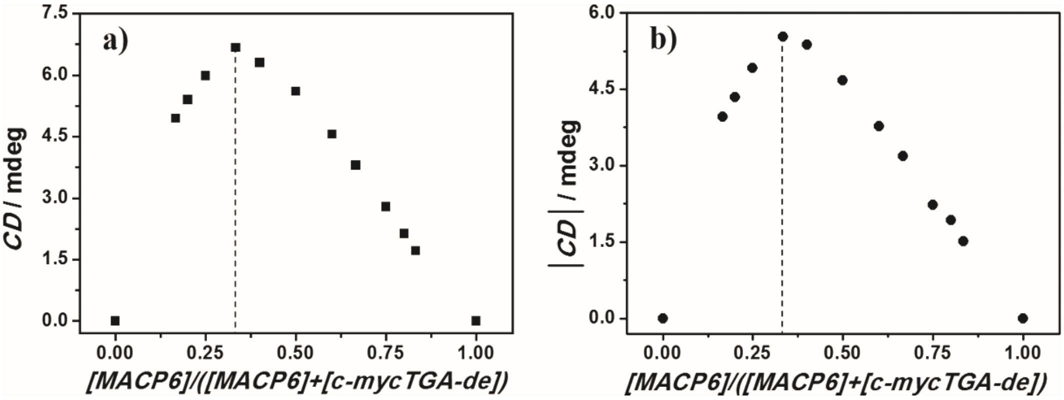

Figure S35. The CD Job plot corresponding to the binding between MACP6 and c-mycTGA-de in 17 mM phosphate buffer solution (5% DMSO) at 20 ℃. [MACP6] + [c-mycTGA-de] = 12 μM. At two wavelength (380 nm (a) and 345 nm (b)), the maximum value of y (defined as the CD intensity value of the MACP6/c-mycTGA-de complex) was found at 0.33, a finding consistent with a 1:2 (MACP6:c-kitG20T) binding stoichiometry.

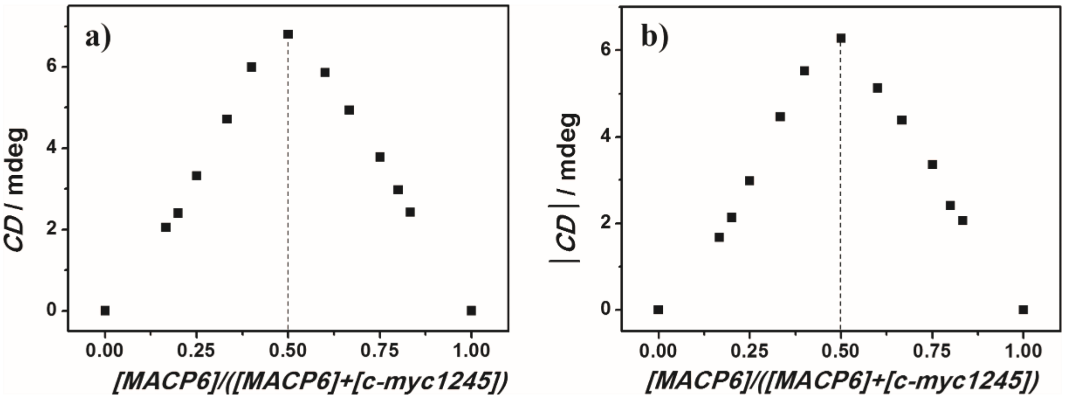

Figure S36. The CD Job plot corresponding to the binding between MACP6 and c-myc1245 in 17 mM phosphate buffer solution (5% DMSO) at 20 ℃. [MACP6] + [c-myc1245] = 12 μM. At two wavelength (380 nm (a) and 345 nm (b)), the maximum value of y (defined as the CD intensity value of the MACP6/c-myc1245 complex) was found at 0.5, a finding consistent with a 1:1 (MACP6:c-myc1245) binding stoichiometry.

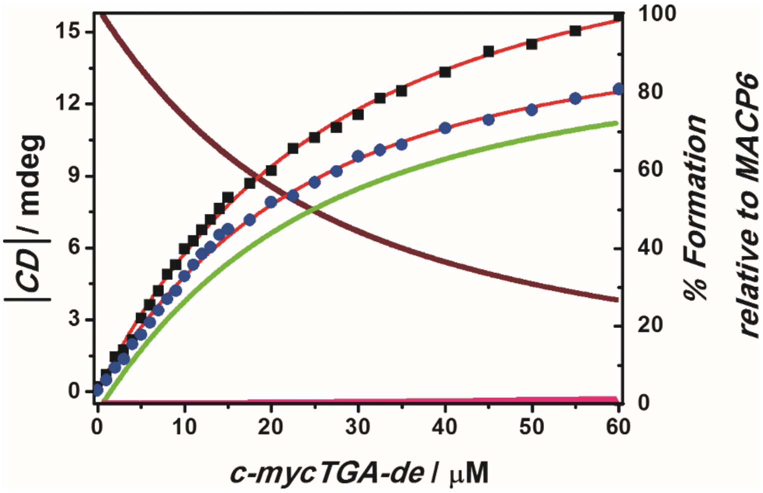

Figure S37. The CD spectra isotherm of 10 μM MACP6 with increasing c-mycTGA-de G-quadruplex (from 0 to 60 μM) in 17 mM phosphate buffer solution (5% DMSO) at 20 ℃ (■ or ● indicate the change at 380 nm or 345 nm in CD spectra of MACP6, respectively). The CD spectra values from 320 nm to 430 nm were used for the calculation of K_{a1}((5.13 ± 0.2) × 10^4 M^{-1}) and K_{a2}((2.8 ±0.2) × 10^2 M^{-1}) using the Hyperquad 2003 program. The red lines show the least-square nonlinear fitting of the experimental data to the appropriate equations. The wine, green and pink lines show the calculated percentage of compound species of [MACP6], [MACP6·c-mycTGA-de] and [MACP6·(c-mycTGA-de)$_2$] vs. MACP6 at each additional c-mycTGA-de concentration.

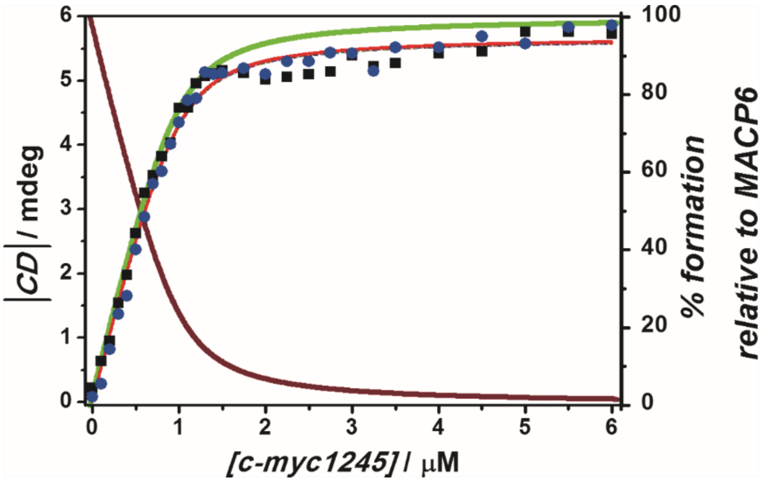

Figure S38. The CD spectra isotherm of 10 μM MACP6 with increasing c-myc1245 G-quadruplex (from 0 to 60 μM) in 17 mM phosphate buffer solution (5% DMSO) at 20 ℃ (■ or ● indicate the change at 380 nm or 345 nm in CD spectra of MACP6, respectively). The CD spectra values from 320 nm to 430 nm were used for the calculation of K_a ((5.1 ± 0.2) × 10^4 M^{-1}) using the Hyperquad 2003 program. The red lines show the least-square nonlinear fitting of the experimental data to the appropriate equations. The wine and green lines show the calculated percentage of compound species of [MACP6] and [MACP6•c-myc1245] vs. MACP6 at each additional c-myc1245 concentration.

3. References

1. Zhang, E.-X., Wang, D.-X., Zheng, Q.-Y. & Wang, M.-X. Synthesis of large macrocyclic azacalix[n]pyridines (n = 6–9) and their complexation with fullerenes C60 and C70. *Org. Lett.* **10**, 2565-2568 (2008).
2. Guan, A. J. *et al.* Effects of loops and nucleotides in G-quadruplexes on their interaction with an azacalixarene, methylazacalix[6]pyridine. *J. Phys. Chem. B* **115**, 12584-12590 (2011).
3. Job, P. Formation and stability of inorganic complexes in solution. *Ann. Chim. Appl.* **9**, 113-203 (1928).

4. Gans, P., Sabatini, A. & Vacca, A. Investigation of equilibria in solution. Determination of equilibrium constants with the HYPERQUAD suite of programs, *Talanta* **43**, 1739-1753 (1996).
5. Haider, S. & Neidle, S. Molecular modeling and simulation of G-quadruplexes and quadruplex-ligand complexes. *Methods Mol Biol* **608**, 17-37 (2010).
